# Supplementary material for: Screening, Synthesis and Biochemical Characterization of SARS-CoV-2 Protease Inhibitors
Source: Int J Mol Sci. 2023 Aug 30;24(17):13491. doi: 10.3390/ijms241713491 (PMC10488051; doi:10.3390/ijms241713491)
Supplement: Supplementary file 1 [file ijms-24-13491-s001.zip › ijms-2540818-supplementary.pdf]

SUPPLEMENTARY MATERIAL

Screening, Synthesis and Biochemical Characterization of SARS-CoV-2 Protease Inhibitors

Martynas Bagdonas [1], Kamilė Čerepenkaitė [1], Aurelija Mickevičiūtė [1], Rūta Kananavičiūtė [2], Birutė Grybaitė [3], Kazimieras Anusevičius [3], Audronė Rukšėnaitė [4], Tautvydas Kojis [1], Marius Gedgaudas [1], Vytautas Mickevičius [3], Daumantas Matulis [1], Asta Zubrienė [1,*] and Jurgita Matulienė [1,*]

[1] Department of Biothermodynamics and Drug Design, Institute of Biotechnology, Life Sciences Center, Vilnius University, Saulėtekio 7, LT-10257 Vilnius, Lithuania
[2] Department of Microbiology and Biotechnology, Institute of Biosciences, Life Sciences Center, Vilnius University, Saulėtekio 7, LT-10257 Vilnius, Lithuania
[3] Department of Organic Chemistry, Kaunas University of Technology, Radvilenų pl. 19, LT-50254 Kaunas, Lithuania
[4] Department of Biological DNA Modification, Institute of Biotechnology, Life Sciences Center, Vilnius University, LT-10257 Vilnius, Lithuania
* Correspondence: asta.zubriene@bti.vu.lt (A.Z.); jurgita.matuliene@bti.vu.lt (J.M.)

TABLE OF CONTENT

1. Synthetic details for the preparation of new compounds.
2. ^{1}H and ^{13}C NMR spectra for new compounds (Supplemental Figures S1–S18).
3. Dose-response curves of PL^{pro} (Supplemental Figure S19).
4. Dose-response curves of M^{pro}(Supplemental Figure S20).
5. Primers used in PL^{pro}C111S mutagenesis (Table S1).
6. Sequencing analysis of PL^{pro} WT and PL^{pro}C111S plasmids.
7. Mass spectra of native PL^{pro} in complex with compound **34** (Supplemental Figure S21).

1. Synthetic details for the preparation of new compounds.

*3-(Naphthalen-1-ylamino)propanoic acid (**1**)*

1-Naphthylamine (20 g, 140 mmol), acrylic acid (12 g, 168 mmol), acetic acid (13 ml), and water (3 ml) were heated for 10 h. Sodium hydroxide solution (10%, 150 ml) was added to the cooled mixture and the residue of amine was extracted with diethyl ether (3 x 50 ml). The basic solution was acidified with acetic acid to pH 6. Obtained product **1** were filtered off, and washed with water, hexane and dried. Yield 9.45 g (31%), white solid, m. p. 190–191 °C. ^{1}H NMR (400 MHz, DMSO-*d6*) δ: 2.68 (t, *J* = 7.0 Hz, 2H, CH_2CO); 3.46 (t, *J* = 7.0 Hz, 2H, NCH_2); 6.15 (br. s., 1H, NH); 6.54 (d, *J* = 7.7 Hz, 1H, H_{Ar}); 7.13 (d, *J* = 8.1 Hz, 1H, H_{Ar}); 7.30 (t, *J* = 7.9 Hz, 1H, H_{Ar}); 7.35–7.50 (m, 2H, H_{Ar}); 7.76 (d, *J* = 7.8 Hz, 1H, H_{Ar}); 8.13 (d, *J* = 8.2 Hz, 1H, H_{Ar}); 12.33 (br. s., 1H, COOH). ^{13}C BMR (101 MHz, DMSO-*d6*) δ: 33.37 (CH_2CO); 39.21 (NCH_2); 103.02, 115.67, 121.59, 123.12, 124.00, 125.64, 126.86, 127.97, 134.06, 143.81 (C_{Ar}); 173.44 (COOH). IR (KBr): ν, cm^{-1}: 3340 (NH); 3040 (OH); 1721 (CO). Calcd. for $C_{13}H_{13}NO_2$, %: C 72.54; H 6.09; N 6.51. Found, %: C 72.50; H 6.00; N 6.47.

*3-(1-(Naphthalen-1-yl)thioureido)propanoic acid (**2**)*

A mixture of the β-alanine **1** (3 g, 14 mmol), KSCN (1.75 g, 18 mmol), and acetic acid (10 ml) was heated at reflux for 12 h, acidified with conc. HCl to pH 1and heated at reflux for 20 min, and diluted with water (30 ml). The obtained crystals were filtered off, washed with water, hexane and dried. The crystals were dissolved in the 5% NaOH solution (15 ml) by heating. The basic solution was acidified with acetic acid to pH 6. Obtained product **2** was filtered off, and washed with water, hexane and dried. Yield 1.99 g (52%), white solid, m. p. 171–172 °C. ^{1}H NMR (400 MHz, DMSO-*d6*) δ: 2.53–2.73 (m, 2H, CH_2CO); 3.84 and 4.73 (2m, 2H, NCH_2); 6.33 (br. s., 2H, NH_2); 7.39–7.52 (m, 1H, H_{Ar}); 7.53–7.80 (m, 4H, H_{Ar}); 7.92–8.13 (m, 2H, H_{Ar}); 12.29 (br. s., 1H, COOH). ^{13}C BMR (101 MHz, DMSO-*d6*) δ: 32.68 (CH_2CO); 50.51 (NCH_2); 122.10, 126.30, 126.60, 127.29, 128.68, 128.80, 128.94, 129.05, 134.64, 157.64 (C_{Ar}); 172.47 (COOH); 182.19 ($S=CNH_2$). IR (KBr): ν, cm^{-1}: 3485 (NH); 3441 (OH); 1702 (CO). Calcd. for $C_{14}H_{14}N_2O_2S$, %: C 61.29; H 5.14; N 10.21. Found, %: C 61.50; H 5.42; N 10.50.

*3-(Naphthalen-1-yl(thiazol-2-yl)amino)propanoic acid (**3**)*

Was synthesized according to the described procedure [1].

*3-((4-methylthiazol-2-yl)(naphthalen-1-yl)amino)propanoic acid (**4**)*

Was synthesized according to the described procedure [1].

*3-(naphthalen-1-yl(4-oxo-4,5-dihydrothiazol-2-yl)amino)propanoic acid (**5**)*

Was synthesized according to the described procedure [1].

*3-((4-(4-bromophenyl)thiazol-2-yl)(naphthalen-1-yl)amino)propanoic acid (**6a**)*

Was synthesized according to the described procedure [1].

*3-((4-(4-chlorophenyl)thiazol-2-yl)(naphthalen-1-yl)amino)propanoic acid (**6b**)*

Was synthesized according to the described procedure [2].

*3-((4-(4-fluorophenyl)thiazol-2-yl)(naphthalen-1-yl)amino)propanoic acid (**6c**)*

Was synthesized according to the described procedure [2].

*3-(naphthalen-1-yl(4-(4-nitrophenyl)thiazol-2-yl)amino)propanoic acid (**6d**)*

Was synthesized according to the described procedure [1].

*3-((4-(4-cyanophenyl)thiazol-2-yl)(naphthalen-1-yl)amino)propanoic acid (**6e**)*

Was synthesized according to the described procedure [1].

3-((4-(3,4-dichlorophenyl)thiazol-2-yl)(naphthalen-1-yl)amino)propanoic acid (7)

A mixture of thioureido acid **2** (2.3 g, 8.4 mmol), the 2-bromo-3′,4′-dichloroacetophenone (2.79 g, 10.4 mmol) and acetone (40 mL) was refluxed for 3 h. The formed *N*,*N*-disubstituted aminothiazole hydrobromide was filtered off, washed with plenty of acetone and then boiled in 4% aqueous sodium acetate for 5 min. The obtained appropriate product 7 was filtered off, washed with water and dried. Yield 2.23 g (60%), white solid, m. p. 157–158 ºC. ^{1}H BMR (700 MHz, DMSO-d_6) δ: 2.60–2.82 (m, 2H, CH_2CO); 3.97–4.18 and 4.44–4.62 (2m, 2H, NCH_2); 7.31 (s, 1H, SCH); 7.53–7.78 (m, 5H, H_{Ar}); 7.80–7.96 (m, 2H, H_{Ar}) 8.03–8.19 (m, 3H, H_{Ar}). ^{13}C BMR (176 MHz, DMSO-d_6) δ: 32.60 (CH_2CO); 48.78 (NCH_2); 105.49; 122.31; 125.81; 126.49; 126.80; 127.25; 127.30; 127.44; 128.81; 129.21; 129.71; 130.84; 131.40; 134.83; 136.08; 140.11; 147.62 (C_{Ar}, S−CH=C); 170.20 (C=N); 172.62 (COOH). IR (KBr): ν, cm^{-1}: 3048 (OH); 1721 (CO); 1532 (C=N). Calcd. for $C_{22}H_{16}Cl_2N_2O_2S$, %: C 59.60; H 3.64; N 6.32. Found, %: C 59.74; H 3.54; N 6.22.

*3-(naphthalen-1-yl(4-(naphthalen-2-yl)thiazol-2-yl)amino)propanoic acid (**8**)*

Was synthesized according to the described procedure [1].

*3-((4-(chloromethyl)thiazol-2-yl)(naphthalen-1-yl)amino)propanoic acid (**9**)*

Was synthesized according to the described procedure [4].

*3-((4-(hydroxymethyl)thiazol-2-yl)(naphthalen-1-yl)amino)propanoic acid (**10**)*

Was synthesized according to the described procedure [4].

*3-((4-(2-ethoxy-2-oxoethyl)thiazol-2-yl)(naphthalen-1-yl)amino)propanoic acid (**11**)*

Was synthesized according to the described procedure [4].

*3-(naphthalen-1-yl(4-((phenylamino)methyl)thiazol-2-yl)amino)propanoic acid (**12**)*

Was synthesized according to the described procedure [4].

*3-(((5Z)-5-(4-Bromobenzylidene)-4-oxo-4,5-dihydro-1,3-thiazol-2-yl)(naphthalen-1 yl)amino)propanoic acid (**13a**)*

Was synthesized according to the described procedure [1].

*(((5Z)-5-(4-Chlorobenzylidene)-4-oxo-4,5-dihydro-1,3-thiazol-2-yl)(naphthalen-1-yl)amino)propanoic acid (**13b**)*

Was synthesized according to the described procedure [1].

*3-(((5Z)-5-(4-Fluorobenzylidene)-4-oxo-4,5-dihydro-1,3-thiazol-2-yl)(naphthalen-1-yl)amino)propanoic acid (**13c**)*

Was synthesized according to the described procedure [1].

General synthetic procedure for **14−16**.

A mixture of compound **5** (0.57 g, 1.8 mmol), the corresponding aldehyde (1.98 mmol), sodium carbonate (1.01 g, 9.5 mmol), and water (10 mL) was refluxed for 3 h. The cooled reaction mixture was acidified with acetic acid to pH 6, the formed solid was filtered off, washed with water, dried. Purification was performed by dissolving crystals in 5% aqueous sodium carbonate (10 ml H_2O, 0,5 g Na_2CO_3), filtering and acidifying the filtrate with acetic acid to pH 6 to give **14−16** as solids.

*(Z)-3-((5-(2,4-dihydroxybenzylidene)-4-oxo-4,5-dihydrothiazol-2-yl)(naphthalen-1-yl)amino)propanoic acid (**14**)*

Yield 0.58 g (74%), greenish solid, m. p. 205–206 ºC. ^{1}H BMR (700 MHz, DMSO-*d6*) δ: 2.64 (t, 2H, *J* = 7.8 Hz, CH_2CO); 3.87–3.93 and 4.57–4.61 (2m, 2H, NCH_2); 6.24–6.52 (m, 1H, H_{Ar}); 6.61–6.78 (m, 1H, H_{Ar}); 7.22–7.45 (m, 2H, H_{Ar}); 7.55–7.68 (m, 2H, H_{Ar}); 7.72–7.92 (m, 2H, H_{Ar}); 8.06–8.41 (m, 3H, H_{Ar}, CH); 10.16 (s, 1H, OH); 10.27 (s, 1H, OH); 12.24 (br. s., 1H, COOH). ^{13}C BMR (176 MHz, DMSO-*d6*) δ: 32.41 (CH_2CO); 50.32 (NCH_2); 121.86, 122.67, 125.71, 126.17, 126.31, 126.86, 127.07, 127.21, 127.27, 127.68, 128.07, 128.49, 128.89, 129.08, 130.33, 134.27, 136.13, 164.954 (C_{Ar}, SC=CH); 173.43 (COOH); 177.11 (C=N); 179.24 (CO). IR (KBr), ν, cm^{-1}: 3388 (OH); 1692 (CO); 1526 (C=N). Calcd. for $C_{23}H_{18}N_2O_5S$, %: C 63.58; H 4.18; N 6.45. Found, %: C 63.50; H 4.08; N 6.38.

*(Z)-3-((5-(2-hydroxybenzylidene)-4-oxo-4,5-dihydrothiazol-2-yl)(naphthalen-1-yl)amino)propanoic acid (**15**)*

Yield 0.41 g (54 %), greenish solid, m. p. 203–204 ºC. ^{1}H BMR (400 MHz, DMSO-*d6*) δ: 2.73 (t, 2H, *J* = 7.8 Hz, CH_2CO); 3.97–4.04 ir 4.68–4.75 (2m, 2H, NCH_2); 6.70 (t, 2H, *J* = 7.6 Hz, H_{Ar}); 6.85 (d, 2H, *J* = 7.8 Hz, H_{Ar}); 6.96 (t, 2H, *J* = 7.9 Hz, H_{Ar}); 7.14 (t, 2H, *J* = 7.8 Hz, H_{Ar}); 7.49–7.89 (m, 5H, H_{Ar}); 7.92 (s, 1H, CH); 8.05–8.31 (m, 2H, H_{Ar}); 10.31 (s, 1H, OH); 12.42 (pl. s, 1H, COOH). ^{13}C BMR (101 MHz, DMSO-*d6*) δ: 32.16 (CH_2CO); 49.71 (NCH_2); 115.91; 119.53; 120.50; 121.81; 125.88; 126.04; 127.22; 127.77; 127.91; 127.98; 128.28; 128.89; 129.11; 130.72; 131.51; 134.25; 136.80; 156.82 (C_{Ar}, SC=CH); 171.93 (COOH); 177.39 (C=N); 179.80 (CO). IR (KBr), ν, cm^{-1}: 3060 (OH); 1723 (CO); 1534 (C=N). Calcd. for $C_{23}H_{18}N_2O_4S$, %: C 66.02; H 4.34; N 6.69. Found, %: C 66.26; H 4.28; N 6.75.

*(Z)-3-(naphthalen-1-yl(4-oxo-5-(thiophen-2-ylmethylene)-4,5-dihydrothiazol-2-yl)amino)propanoic acid (**16**)*

Yield 0,53 g (72 %), yellowis solid, m. p. 207–208 ºC.^{1}H BMR (400 MHz, DMSO-*d6*) δ: 2.63–2.68 (m, 2H, CH_2CO); 3.95–4.02 and 4.65–4.72 (2m, 2H, NCH_2); 7.10–8.18 (m, 11H, H_{Ar}, CH). ^{13}C BMR (101 MHz, DMSO-*d6*) δ: 32.73 (CH_2CO); 50.23 (NCH_2); 121.79; 123.58; 125.83; 127.21; 127.30; 128.00; 128.28; 128.79; 128.88; 129.09; 130.75; 131.36; 133.31; 134.22; 135.80; 138.25 (C_{Ar}, SC=CH); 172.19 (COOH); 176.21 (CO); 179.29 (C=N). IR (KBr), ν, cm^{-1}: 3397 (OH); 1725 (CO); 1526 (C=N). Calcd. for $C_{21}H_{16}N_2O_3S_2$, %: C 61.75; H 3.95; N 6.86. Found, %: C 61.61; H 3.88; N 6.58.

*3-((5-(2-Methoxy-2-oxoethylidene)-4-oxo-4,5-dihydrothiazol-2-yl)(naphthalen-1-yl)amino)propanoic acid (**17**)*

Was synthesized according to the described procedure [4].

*2-((2-Carboxyethyl)(naphthalen-1-yl)amino)-4-methylthiazole-5-carboxylic acid (**18**)*

Was synthesized according to the described procedure [4].

*3-[(5-Acetyl-4-methyl-1,3-thiazol-2-yl)(naphthalen-1-yl)amino]propanoic acid (**19**)*

Was synthesized according to the described procedure [1].

3-((5-(Ethoxycarbonyl)-4-methylthiazol-2-yl)(naphthalen-1-yl)amino)propanoic acid (**20**)

Was synthesized according to the described procedure [4].

*N-(4-Methyl-5-((E)-3-phenyl-2-propenoyl)-1,3-thiazol-2-yl)-N-(1-naphthyl)-β-alanine (**21a**)*

Was synthesized according to the described procedure [4].

*N-(5-((E)-3-(4-Chlorophenyl)-2-propenoyl)-4-methyl-1,3-thiazol-2-yl)-N-(1-naphthyl)-β-alanine (**21b**)*

Was synthesized according to the described procedure [1].

*N-(5-((E)-3-(4-Fluorophenyl)-2-propenoyl)-4-methyl-1,3-thiazol-2-yl)-N-(1-naphthyl)-β-alanine (**21c**)*

Was synthesized according to the described procedure [1].

*(E)-3-((4-methyl-5-(3-(naphthalen-1-yl)acryloyl)thiazol-2-yl)(naphthalen-1-yl)amino)propanoic acid (**22**)*

To a solution of compound **19** (0.2 g, 0.56 mmol) in 10% NaOH (10 mL) 2-propanol (10 mL) was poured and then 1-naphthaldehyde (0.11 g, 0.67 mmol) was added dropwise. The reaction mixture was heated under reflux for 3 h, then diluted with water (10 mL) and acidified with 30% acetic acid to pH 6. The formed precipitate was filtered off, washed with water, dried and purified by column chromatography. Eluent – chloroform : methanol 100 : 5; R_f = 0.58. Yield 0.14 g (50%), yellow solid, m. p. 111−112 °C. ^{1}H NMR (400 MHz, DMSO-*d*$_6$) δ: 2.59–2.79 (m, 5H, CH_2CO, CH_3); 3.98–4.10 and 4.48–4.61 (2m, 2H, NCH_2); 7.20 and 7.23 (2s, 1H, CHCO); 7.37–7.87 (m, 15H, H_{Ar}, C<u>H</u>=CHCO); 12.43 (br. s., 1H, COOH). ^{13}C NMR (101 MHz, DMSO-*d*$_6$) δ: 19.17 (CH_3); 32.51 (CH_2CO); 48.54 (NCH_2); 121.98 (CCH); 122.79, 122.92, 124.12, 125.29, 125.69, 126.48, 127.11, 127.73, 128.72, 128.90, 129.04, 129.72, 130.47, 131.02, 131.17, 133.28, 134.81, 136.80, 137.68, 139.03 (CAr); 158.96 (SC=C); 171.52 (C=N); 172.35 (COOH); 180.19 (CHCO). IR (KBr), ν, cm^{-1}: 3058 (OH); 1721 (C=O); 1639 (C=O); 1519 (C=N). Calcd. for $C_{30}H_{24}N_2O_3S$, %: C 73.15; H 4.91; N 5.69. Found, %: C 73.10; H 4.88; N 5.65.

*(Z)-3-((4-methyl-5-(1-(2-phenylhydrazineylidene)ethyl)thiazol-2-yl)(naphthalen-1-yl)amino)propanoic acid (**23**)*

A mixture of compound **19** (0.5 g, 1.4 mmol), phenylhydrazine (0.46 g, 4.2 mmol) and 3 drops of glacial acetic acid in methanol (30 mL) was refluxed for 5 h. Then the reaction mixture was cooled down to room temperature and diluted with water (20 mL). The precipitate was filtered off, washed with water, dried and purified by dissolving it in 5% aqueous sodium carbonate solution (2.5 g Na_2CO_3, 47.5 mL H_2O), filtering the solution and acidifying the filtrate with glacial acetic acid to pH 6. The obtained precipitate was filtered off, washed with water and dried. Yield 0.52 g (83%), yellow solid, m. p. 207–208°C. ^{1}H BMR (400 MHz, DMSO-*d*$_6$) δ: 2.13 (s, 3H, CH_3C=N-N); 2.43 (s, 3H, CH_3); 2.67 (t, 2H, J = 7.4 Hz, CH_2CO); 3.80–4.07 ir 4.32–4.56 (2m, 2H, NCH_2); 6.64 (t, 1H, J = 7.3 Hz, H_{Ar}); 6.64 (d, 2H, J = 8.0 Hz, H_{Ar}); 7.09 (t, 2H, J = 7.7 Hz, H_{Ar}); 7.54–7.69 (m, 4H, H_{Ar}); 7.76–7.85 (m, 1H, H_{Ar}); 8.01–8.13 (m, 2H, H_{Ar}); 8.98 (s, 1H, NH); 12,27 (s, 1H, COOH). ^{13}C BMR (101 MHz, DMSO-*d*$_6$) δ: 15.69 (CH_3); 18.24 (CH_3C=N-N); 32.60 (CH_2CO); 48.12 (NCH_2); 118.40; 122.30; 122.33; 126.43; 126.75; 127.29; 127.39; 128.79; 129.04; 129.77; 134.77; 137.77; 140.06 (C_{Ar}); 144.81 (SC=C); 112.35 (<u>C</u>CH_3); 145.83 (C=N); 166.62 ($NCCH_3$); 172.54 (COOH). IR (KBr), ν, cm^{-1}: 3054 (OH); 1712 (CO); 1512 (C=N). Calcd. for $C_{25}H_{24}N_4O_2S$, %: C 67.55; H 5.44; N 12.60. Found, %: C 67.54; H 5.45; N 12.65.

*3,3'-((((1Z,1'Z)-hydrazine-1,2-diylidenebis(ethan-1-yl-1-ylidene))bis(4-methylthiazole-5,2-diyl))bis(naphthalen-1-ylazanediyl))dipropionic acid (**24**)*

A mixture of compound **19** (0.5 g, 1.4 mmol), hydrazine monohydrate (0.42 g, 8.4 mmol) and 3 drops of glacial acetic acid in methanol (20 mL) was refluxed for 5 h. Then the reaction mixture was cooled down to room temperature and diluted with water (30 mL). The precipitate was filtered off, washed

with water, dried and purified by dissolving it in 5% aqueous sodium carbonate solution (2.5 g Na_2CO_3, 47.5 mL H_2O), filtering the solution and acidifying the filtrate with glacial acetic acid to pH 6. The obtained precipitate was filtered off, washed with water and dried. Yield 0.4 g (40%), yellow solid, m. p. 247–248 ºC. ^{1}H BMR (400 MHz, DMSO-d_6) δ: 2.06 (s, 6H, 2x CH_3C=N-N); 2.41 (s, 6H, 2x CCH_3); 2.65 (t, 4H, J = 7.3 Hz, 2x CH_2CO); 3.87–3.94 and 4.40–4.47 (2m, 4H, 2x NCH_2); 7.50–7.66 (m, 8H, H_{Ar}); 7.70–7.81 (m, 2H, H_{Ar}); 7.98–8.11 (m, 4H, H_{Ar}); 12.19 (s, 2H, 2x COOH). ^{13}C BMR (101 MHz, DMSO-d_6) δ: 16.11 (CH_3); 18.76 (CH_3C=N-N); 32.58 (CH_2CO); 48.10 (NCH_2); 122.13; 122.19; 126.40; 126.78; 127.23; 127.47; 128.79; 129.22; 129.47; 134.72; 139.63; 150.30 (C_{Ar}) 156.57 ($NCCH_3$); 168.61 (C=N); 172.42 (COOH). IR (KBr), ν, cm^{-1}: 3057 (OH); 1706 (CO); 1516 (C=N). Calcd. for $C_{38}H_{36}N_6O_4S_2$, %: C 64.75; H 5.15; N 11.92. Found, %: C 64.80; H 5.03; N 11.98.

*3-((5-Cinnamoyl-4-methylthiazol-2-yl)(p-tolyl)amino)propanoic acid **(25a)***

Was synthesized according to the described procedure [5].

*3-((5-(3-(4-Chlorophenyl)acryloyl)-4-methylthiazol-2-yl)(p-tolyl)amino)propanoic acid **(25b)***

Was synthesized according to the described procedure [5].

*3-((5-(3-(4-Fluorophenyl)acryloyl)-4-methylthiazol-2-yl)(p-tolyl)amino)propanoic acid **(25c)***

Was synthesized according to the described procedure [5].

*3-(Naphthalen-1-yl(4-((phenylamino)methyl)thiazol-2-yl)amino)propanoic acid **(26)***

Was synthesized according to the described procedure [4].

*3-((4-(4-Chlorophenyl)-5-((2-phenylhydrazono)methyl)thiazol-2-yl)(p-tolyl)amino)propanoic acid **(27a)***

Was synthesized according to the described procedure [4].

*3-((4-(4-Fluorophenyl)-5-((2-phenylhydrazono)methyl)thiazol-2-yl)(p-tolyl)amino)propanoic acid **(27b)***

Was synthesized according to the described procedure [4].

*3-((4-(4-Cyanophenyl)-5-((2-phenylhydrazono)methyl)thiazol-2-yl)(p-tolyl)amino)propanoic acid **(27c)***

Was synthesized according to the described procedure [4].

*3-((5-((2-Carbamothioylhydrazono)methyl)-4-(4-cyanophenyl)thiazol-2-yl)(p-tolyl)amino)propanoic acid **(28)***

Was synthesized according to the described procedure [4].

*3-((4-(4-Cyanophenyl)-5-formylthiazol-2-yl)(p-tolyl)amino)propanoic acid **(29)***

Was synthesized according to the described procedure [4].

*3-((4-Aminophenyl)(4-(4-chlorophenyl)-1,3-thiazol-2-yl)amino)propanoic acid **(30a)***

Was synthesized according to the described procedure [6].

*3-{(4-Aminophenyl)[4-(4-fluorophenyl)-1,3-thiazol-2-yl]amino}propanoic acid **(30b)***

Was synthesized according to the described procedure [6].

*3-{(4-Aminophenyl)[4-(4-cianophenyl)-1,3-thiazol-2-yl]amino}propanoic acid **(30c)***

Was synthesized according to the described procedure [6].

*3-((4-Aminophenyl){4-[4-(trifluoromethyl)phenyl]thiazol-2-yl}amino)propanoic acid **(30d)***

Was synthesized according to the described procedure [6].

*N-(1-Naphthyl)-N-(4-((E)-2-phenylethyl)-5-((E)-3-phenyl-2-propenoyl)-1,3-thiazol-2-yl)-β-alanine **(31)***

Was synthesized according to the described procedure [1].
The synthesis and characterization of disulfides **32-38** are described in the paper submitted recently [7]

2. 1H and 13C NMR spectra for new compounds

Supplemental Figures S1–S18, representing ^{1}H and ^{13}C NMR spectra of the newly synthesized compounds **1**, **2,** **7, 14–16, 22–24**.

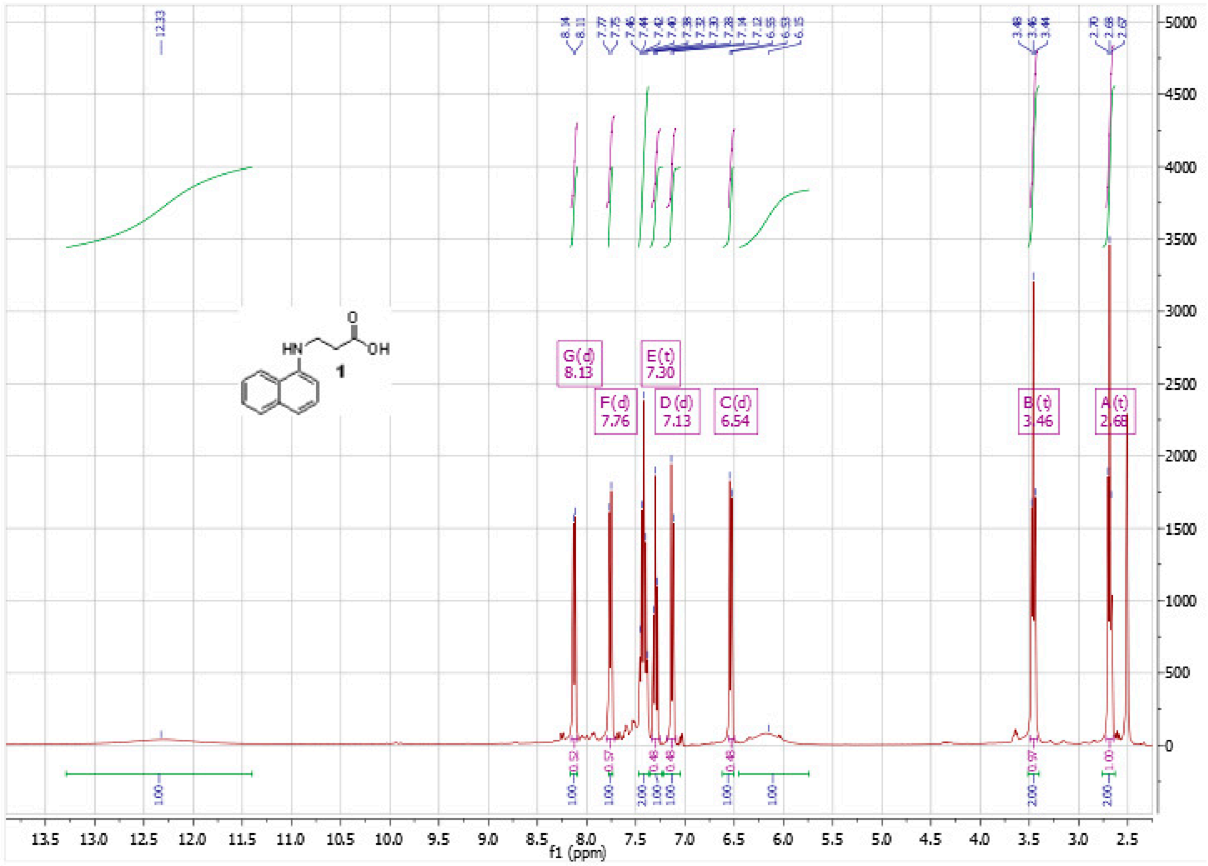

Figure S1: ^{1}H-NMR spectrum of compound **1**.

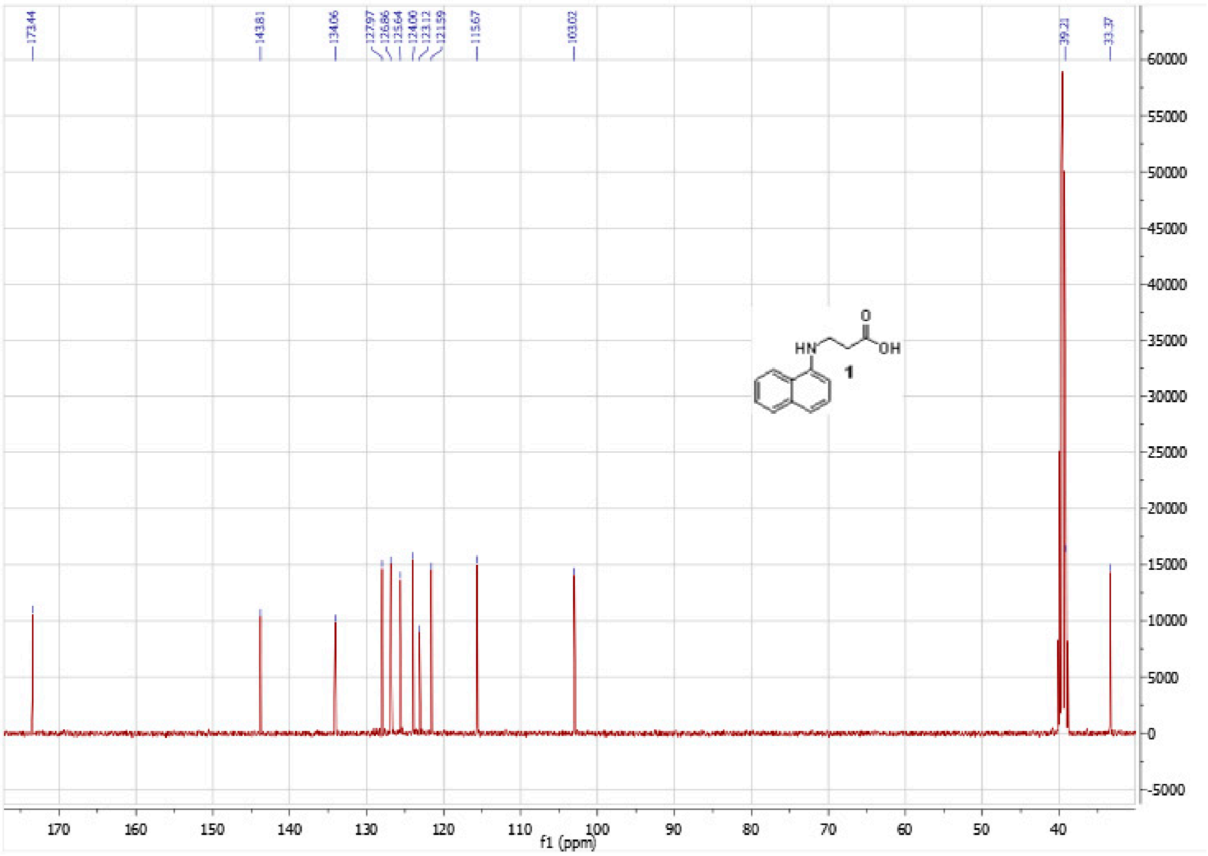

Figure S2: ^{13}C-NMR spectrum of compound **1**.

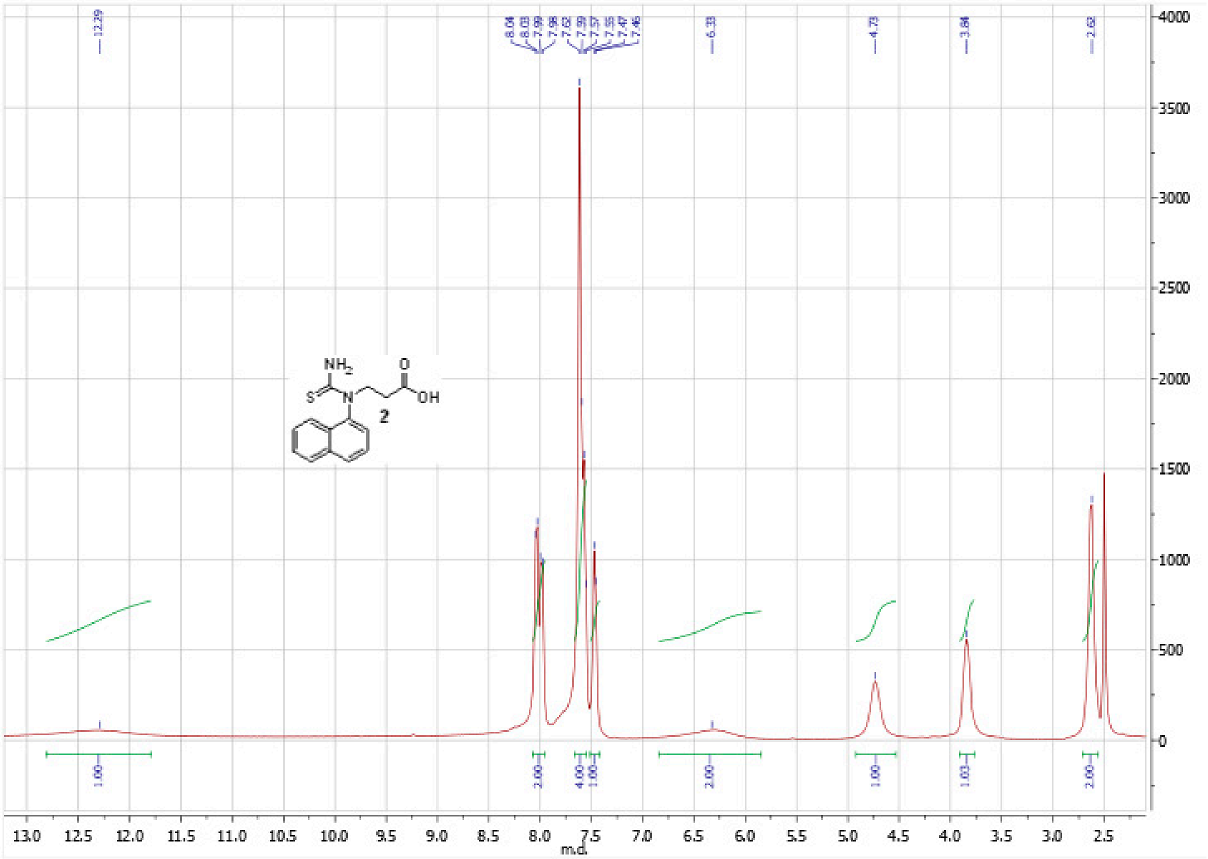

Figure S3: ^{1}H-NMR spectrum of compound **2**.

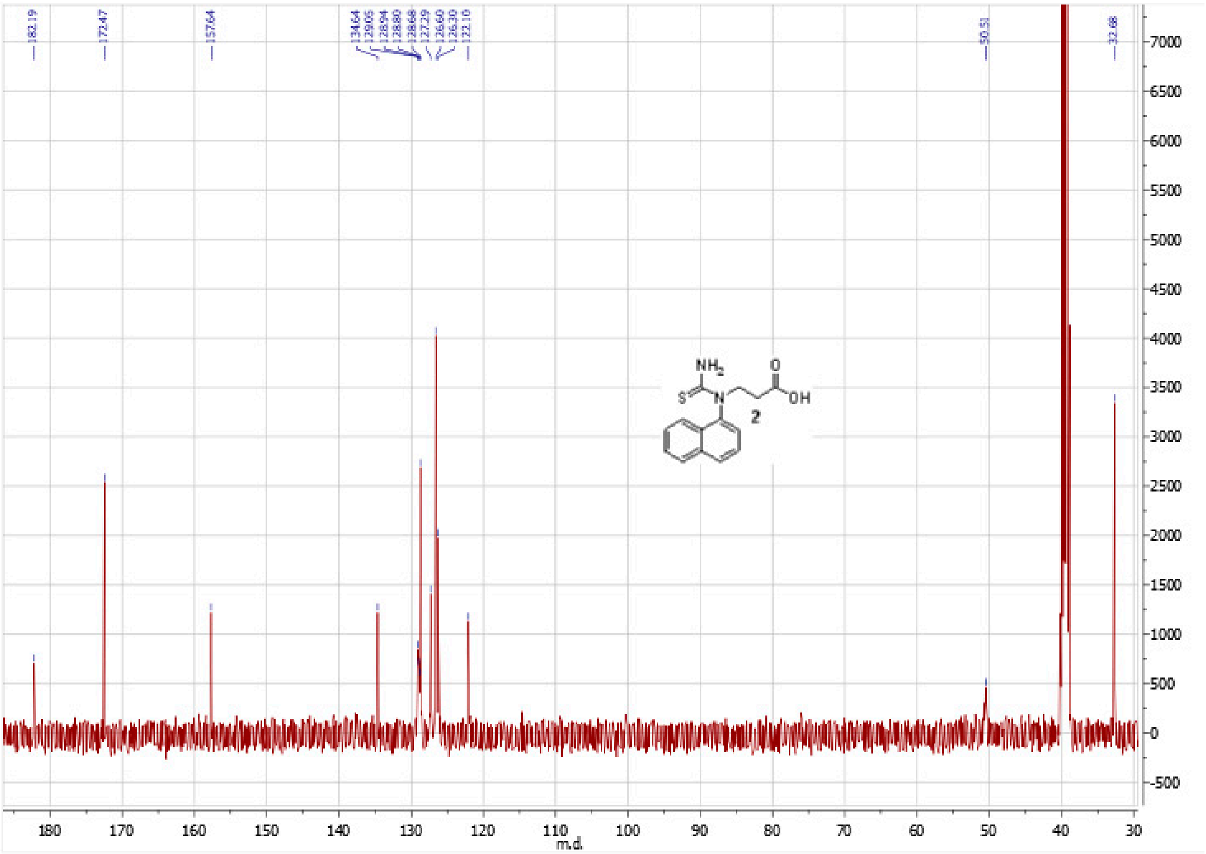

Figure S4: ^{13}C-NMR spectrum of compound **2**.

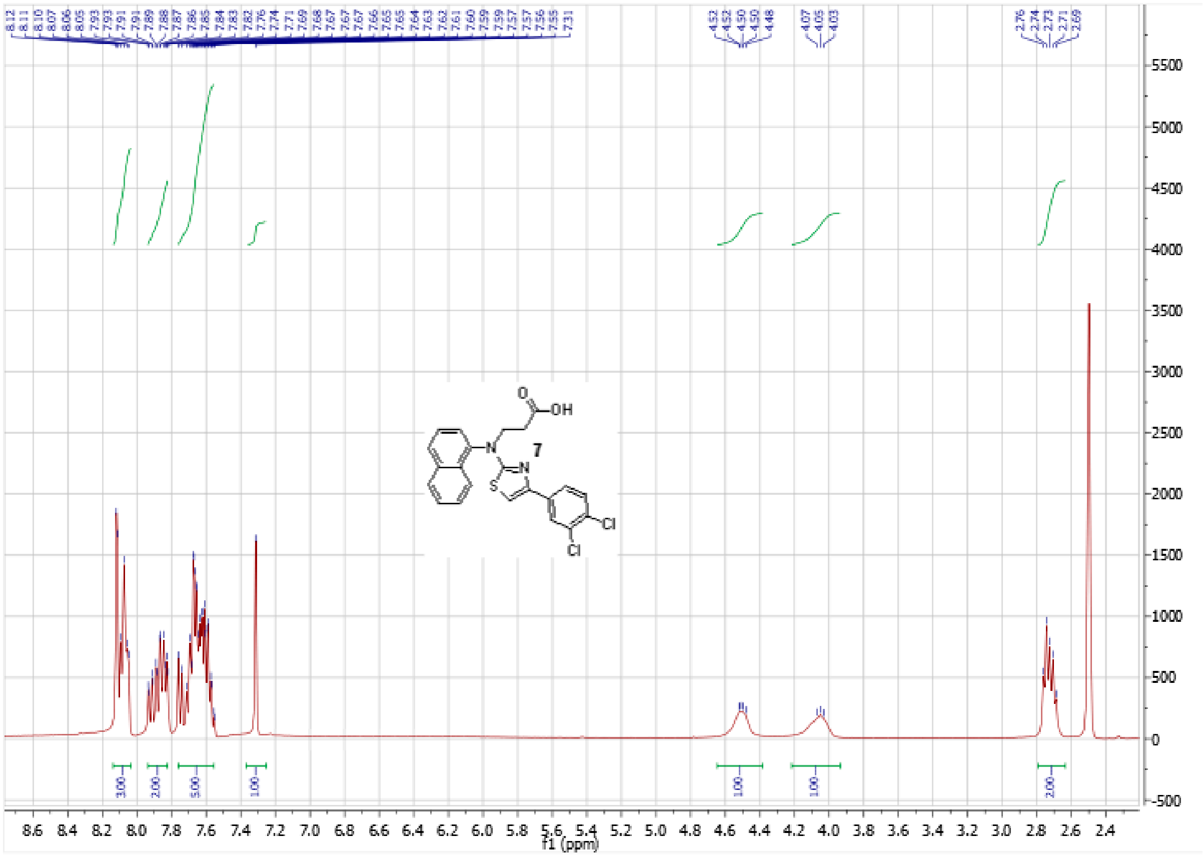

Figure S5: ^{1}H-NMR spectrum of compound **7**.

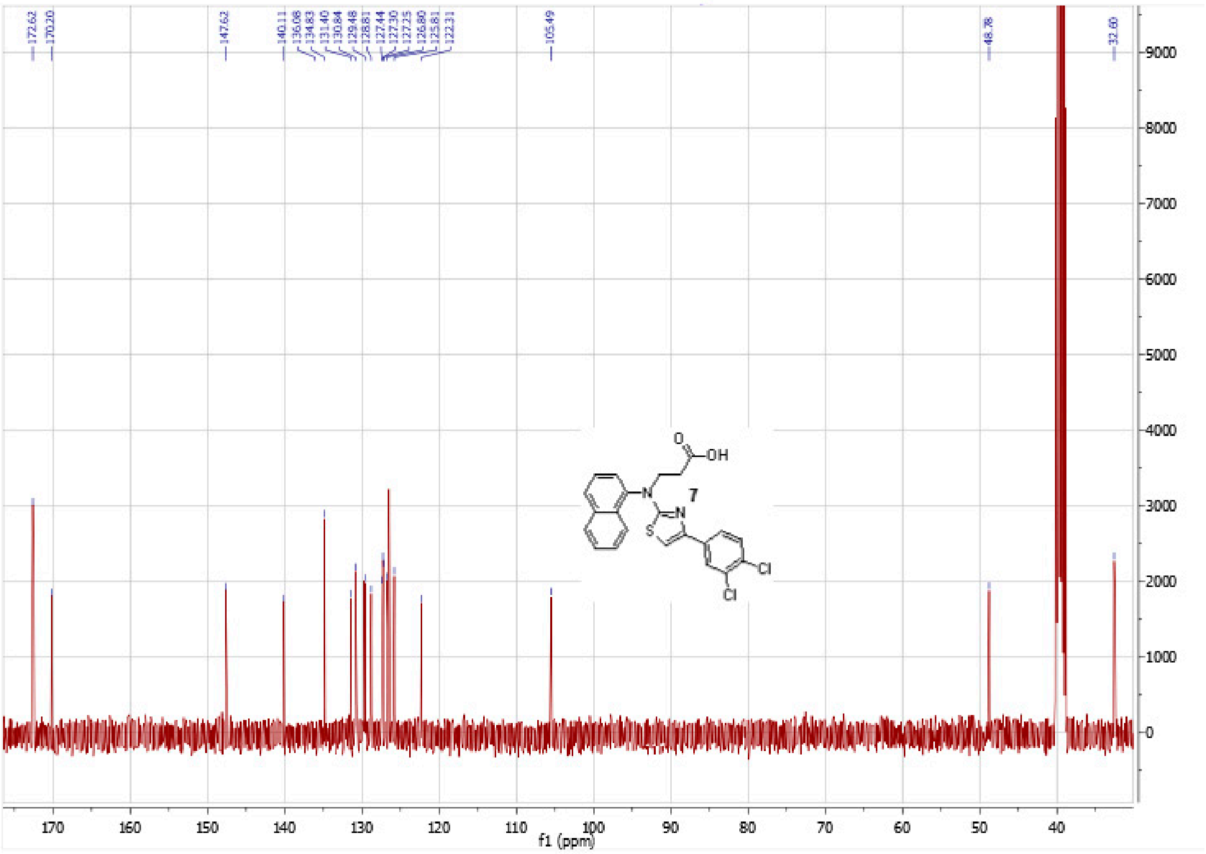

Figure S6: ^{13}C-NMR spectrum of compound **7**.

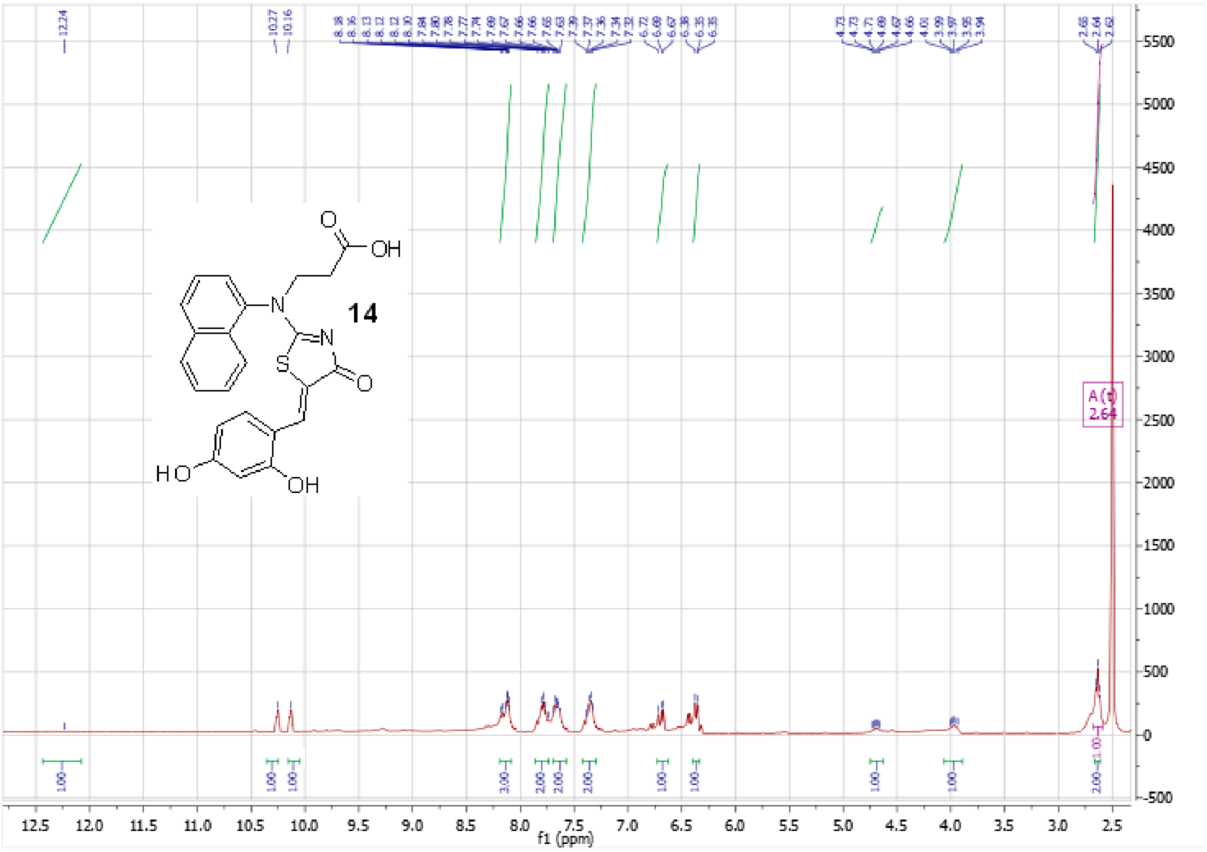

Figure S7: ^{1}H-NMR spectrum of compound **14**.

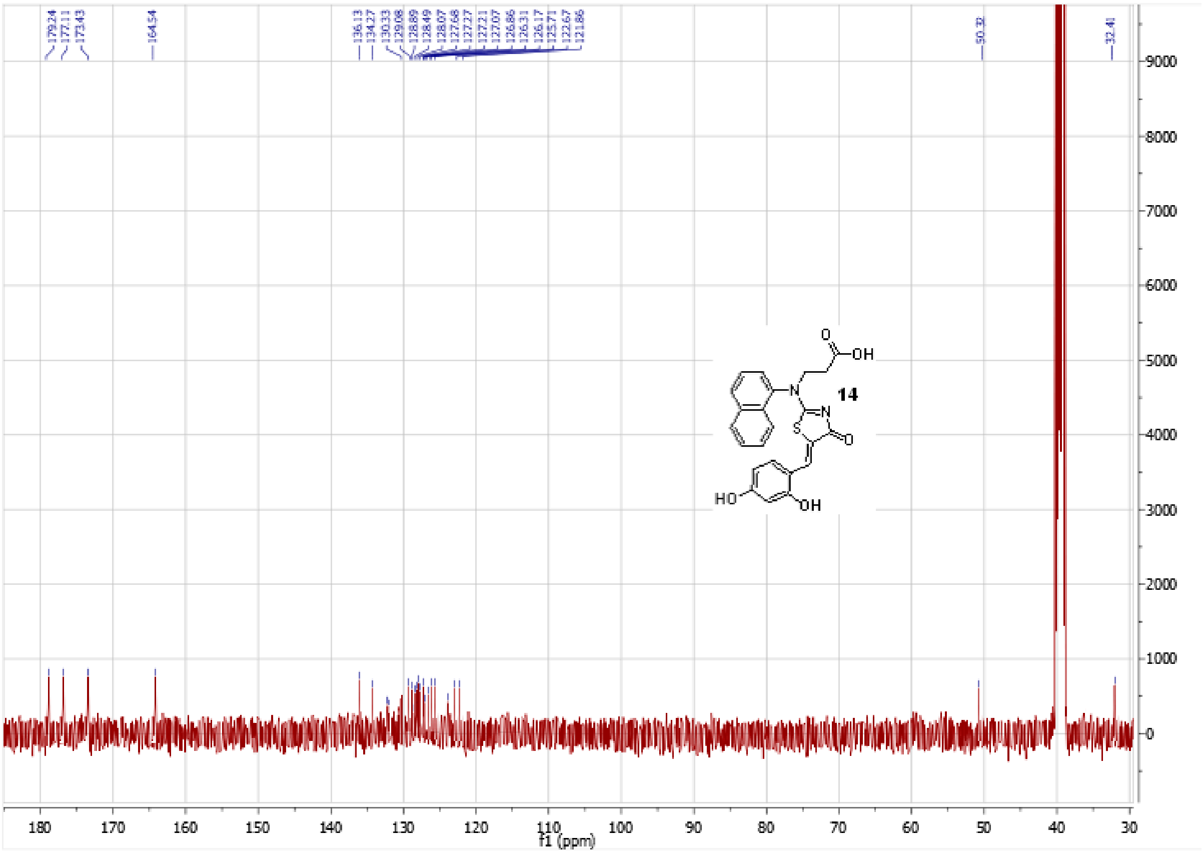

Figure S8: ^{13}C-NMR spectrum of compound **14**.

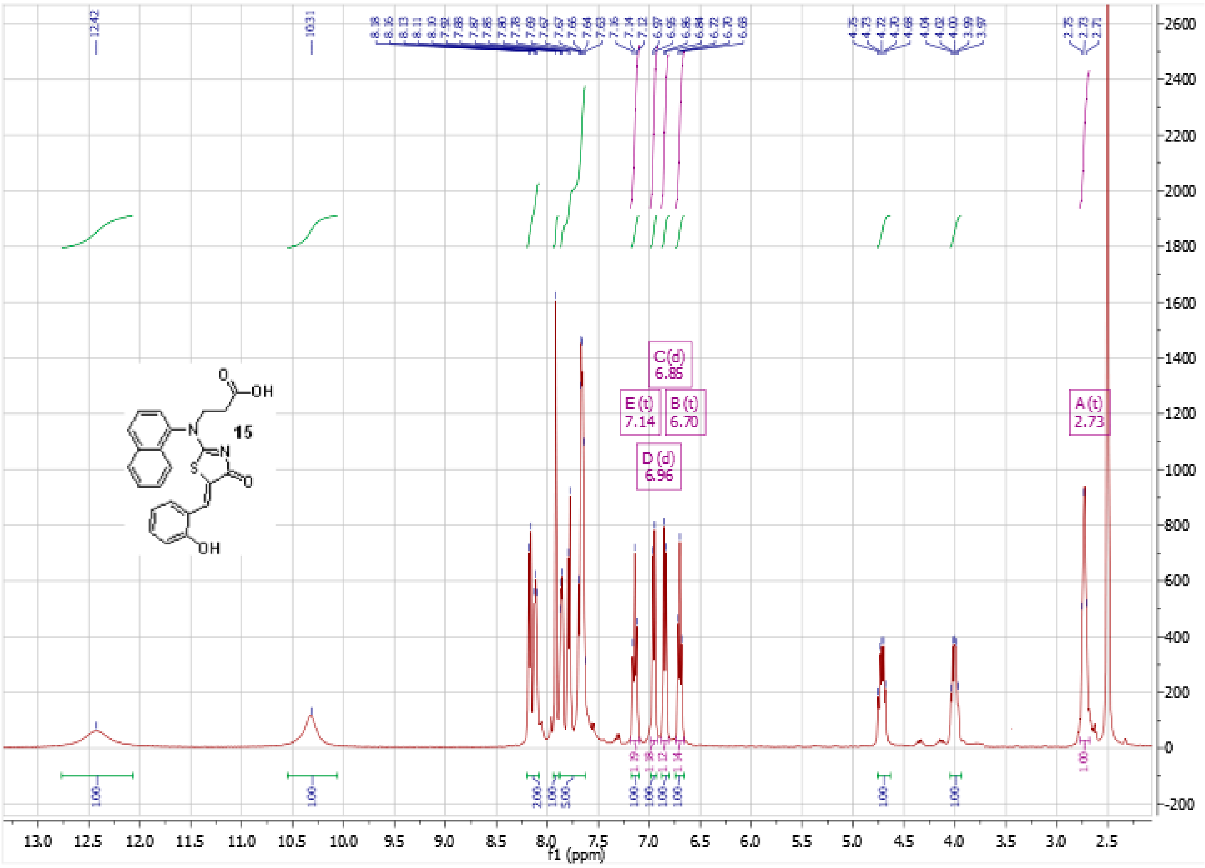

Figure S9: ^{1}H-NMR spectrum of compound **15**.

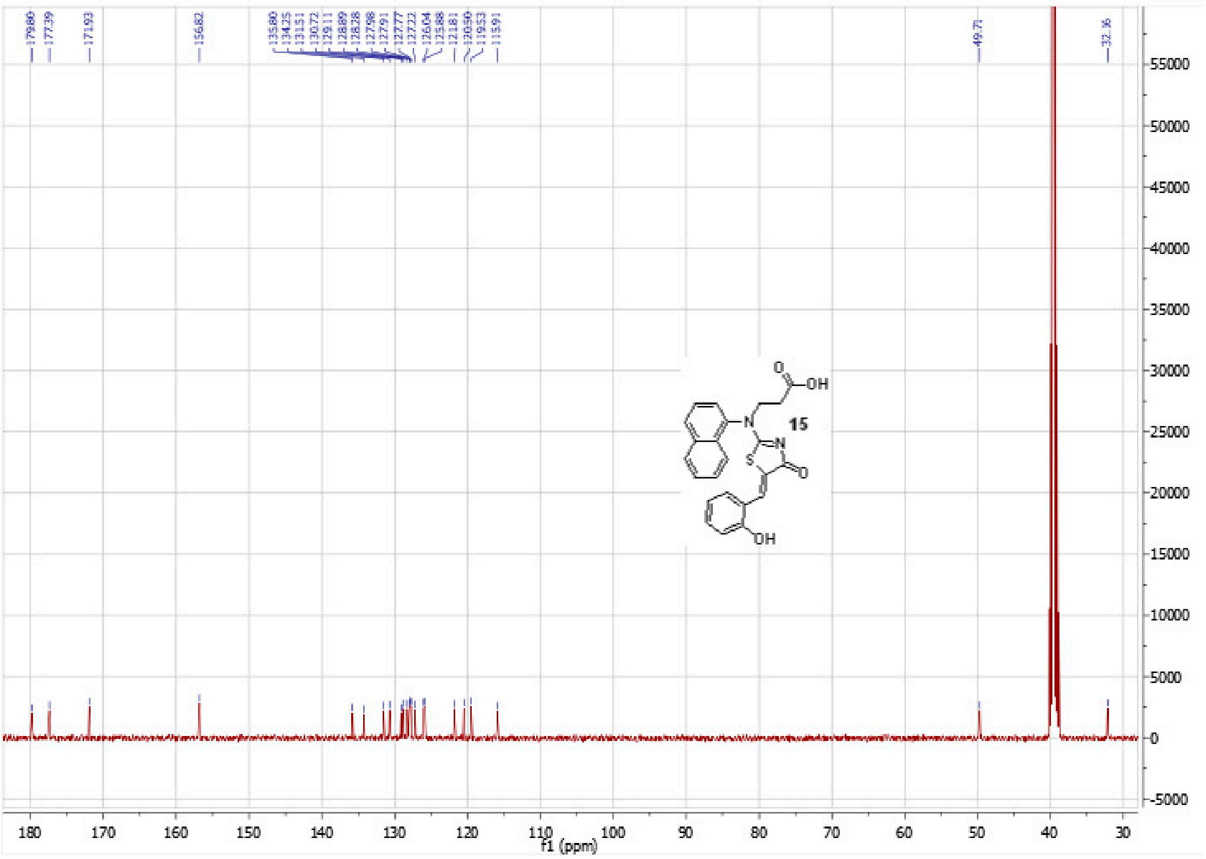

Figure S10: ^{13}C-NMR spectrum of compound **15**.

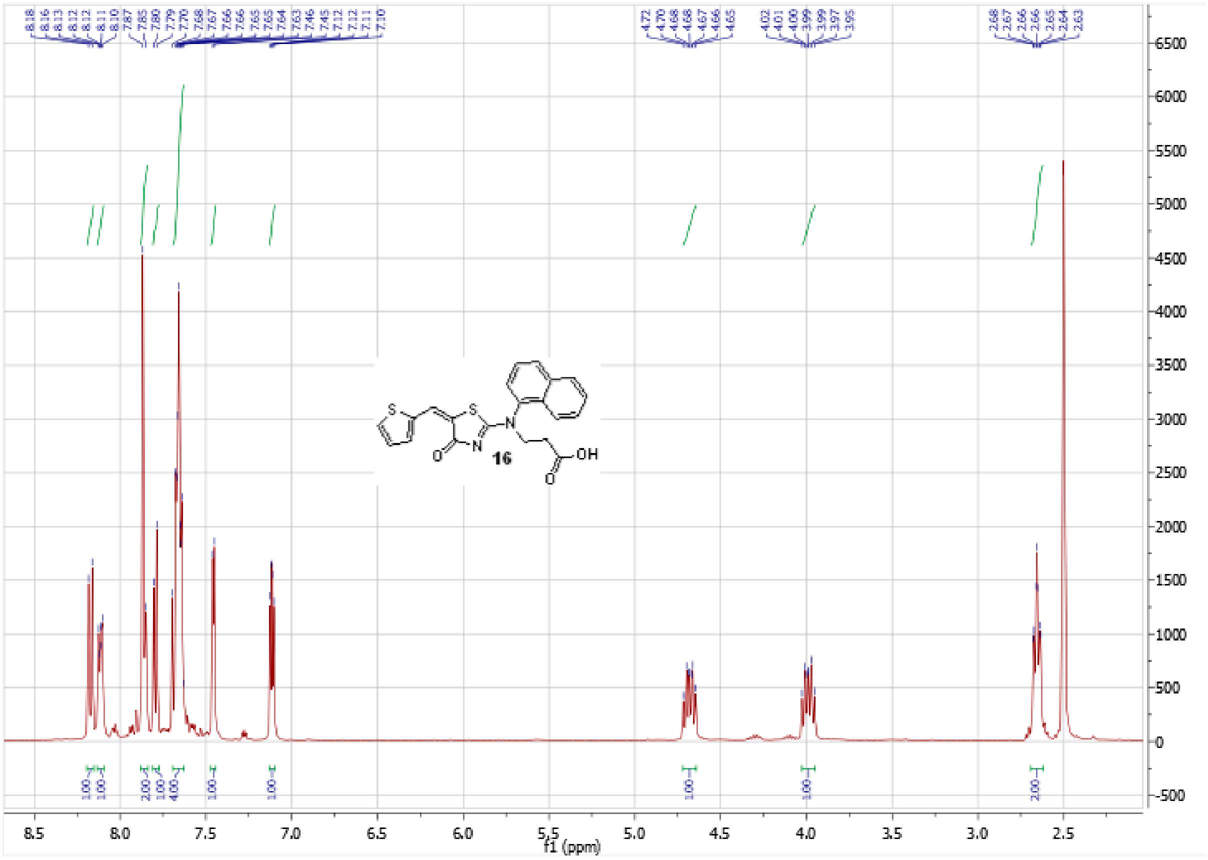

Figure S11: ^{1}H-NMR spectrum of compound **16**.

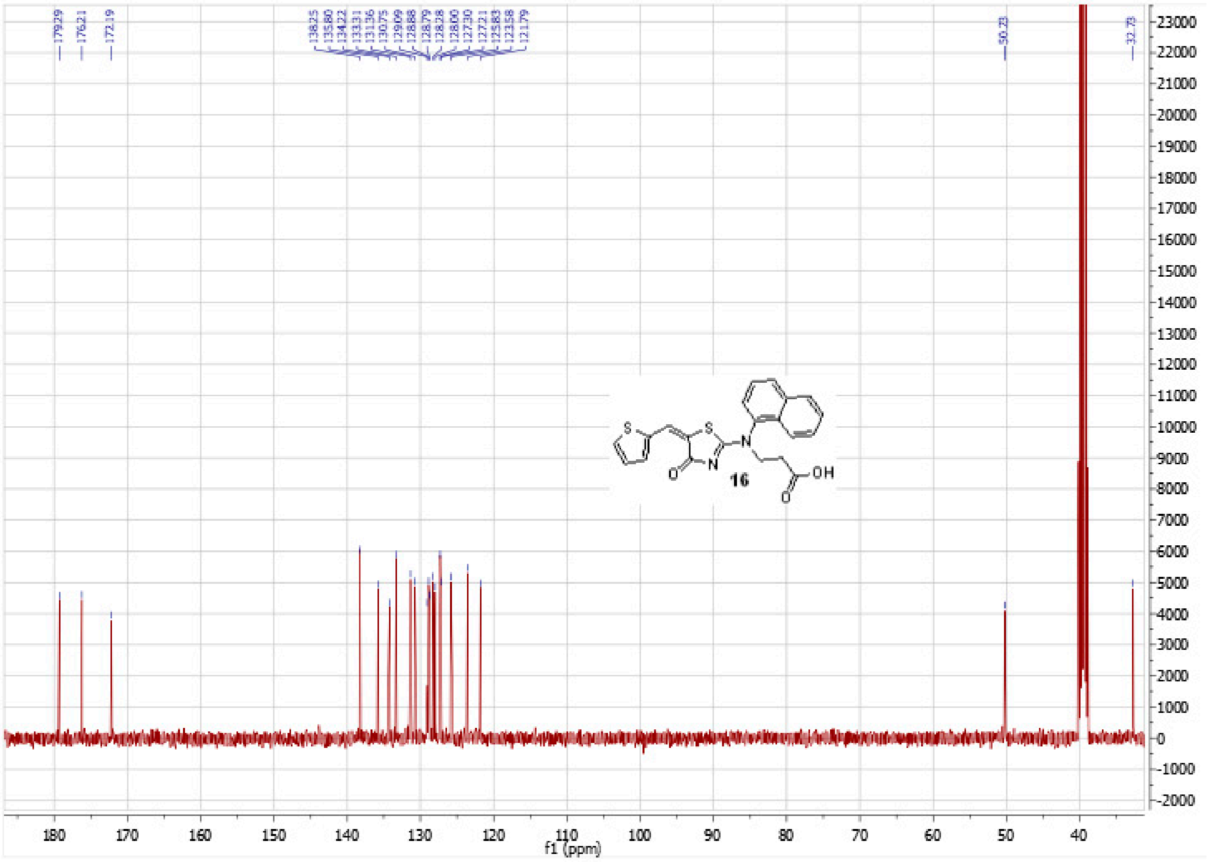

Figure S12: ^{13}C-NMR spectrum of compound **16**.

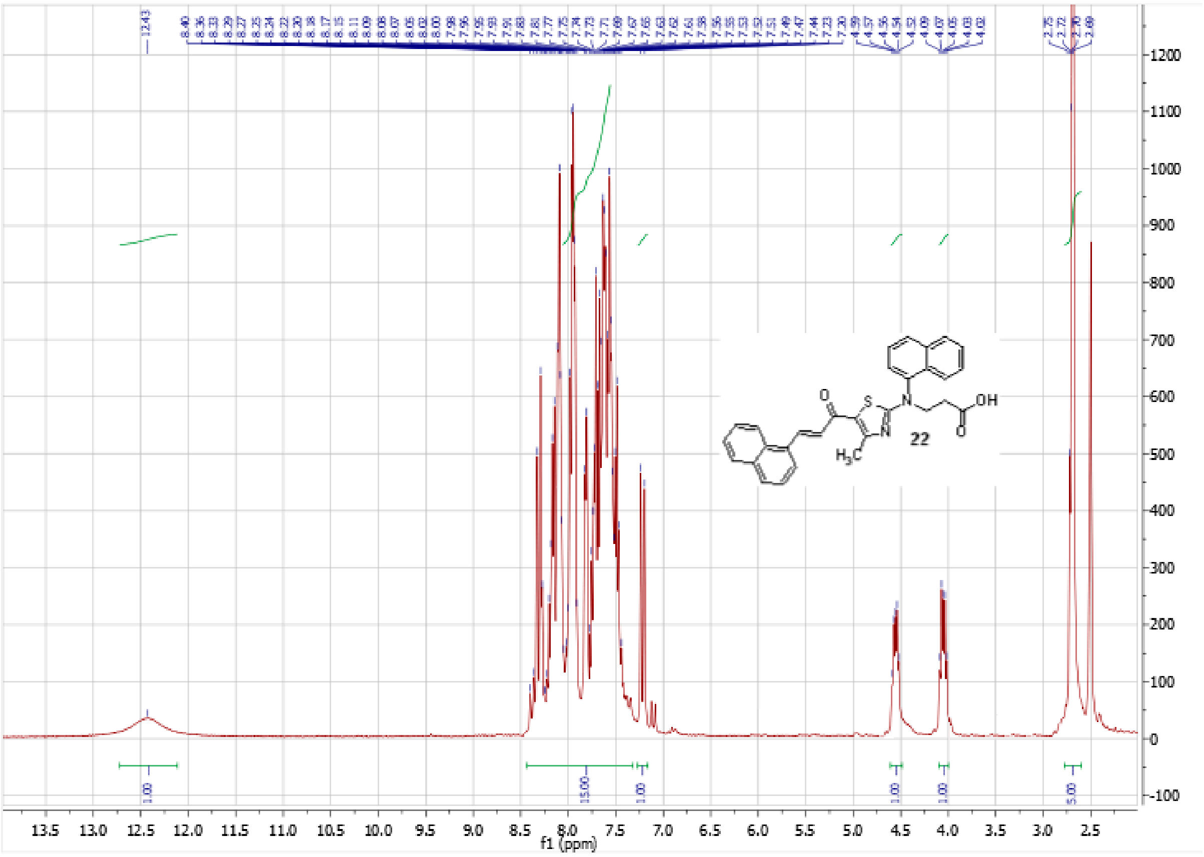

Figure S13: ^{1}H-NMR spectrum of compound **22**.

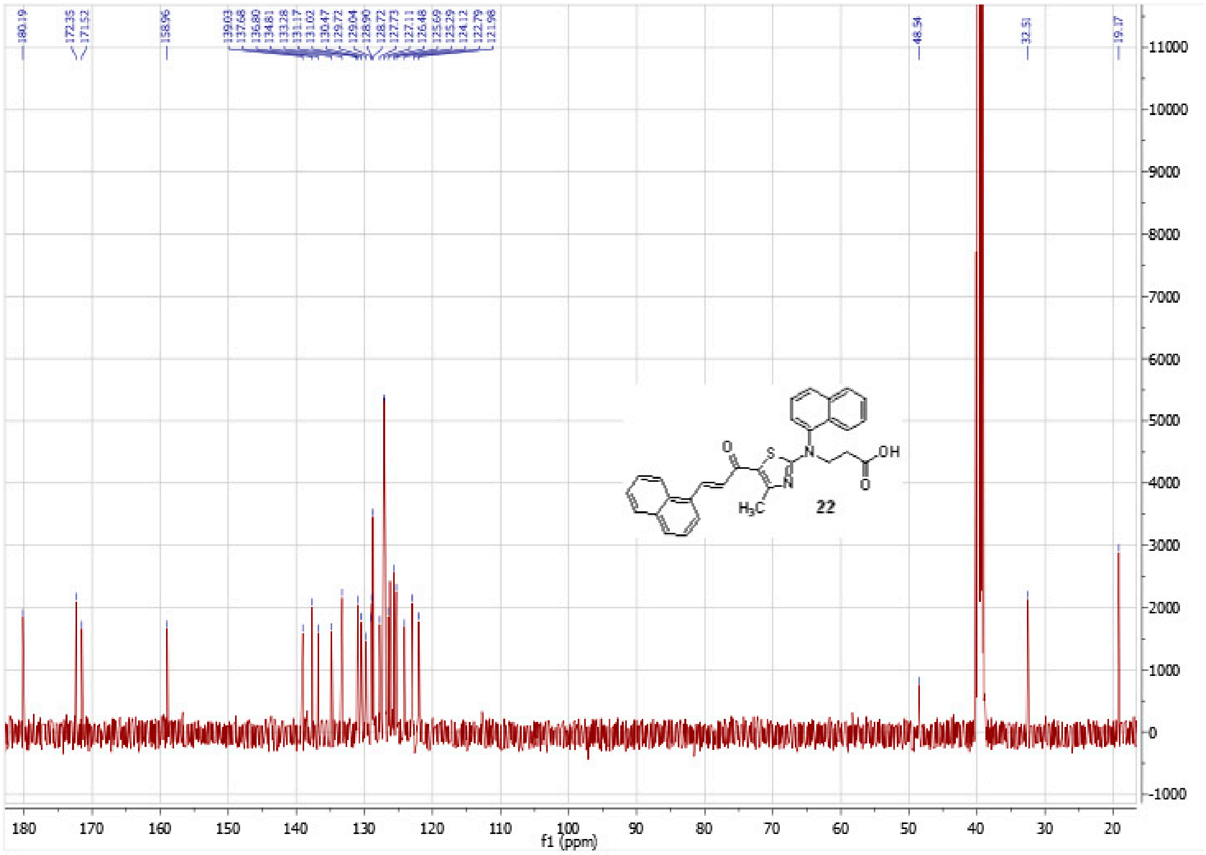

Figure S14: ^{13}C-NMR spectrum of compound **22**.

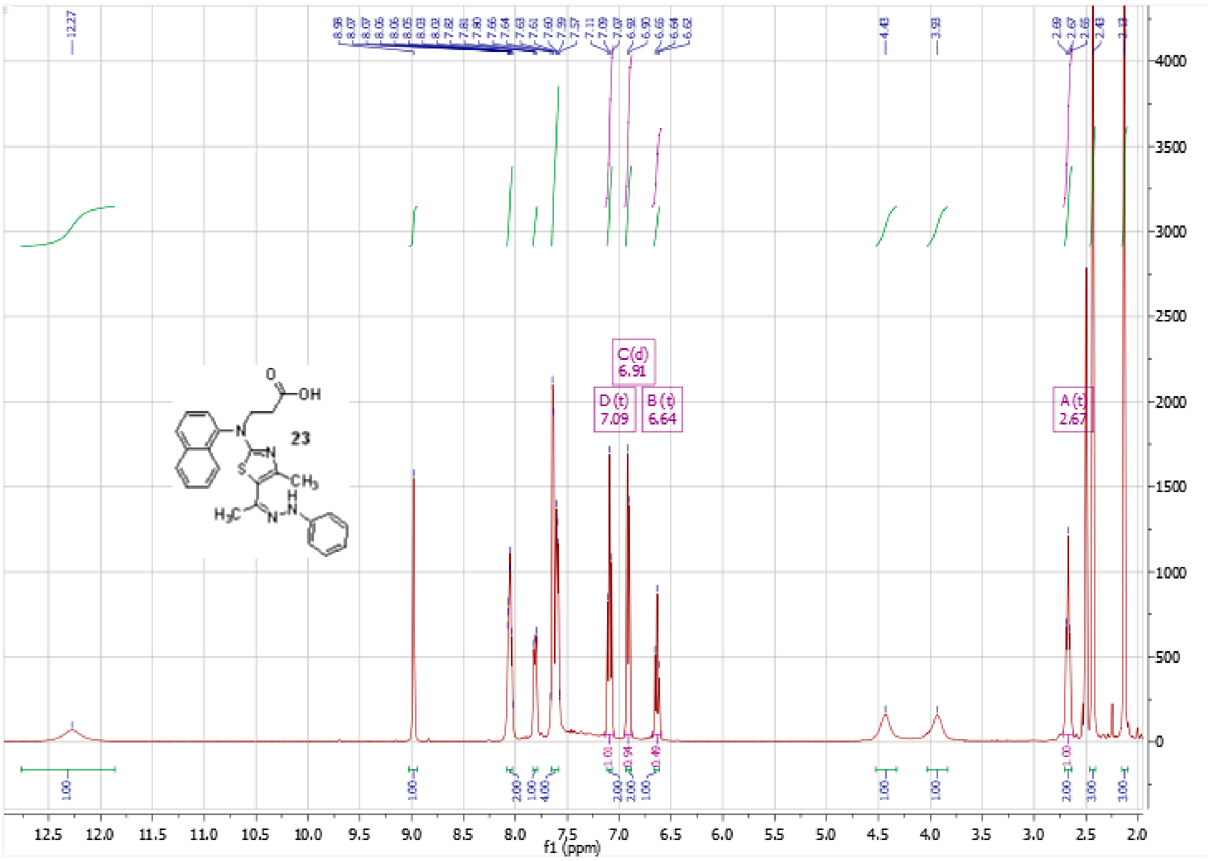

Figure S15: ^{1}H-NMR spectrum of compound **23**.

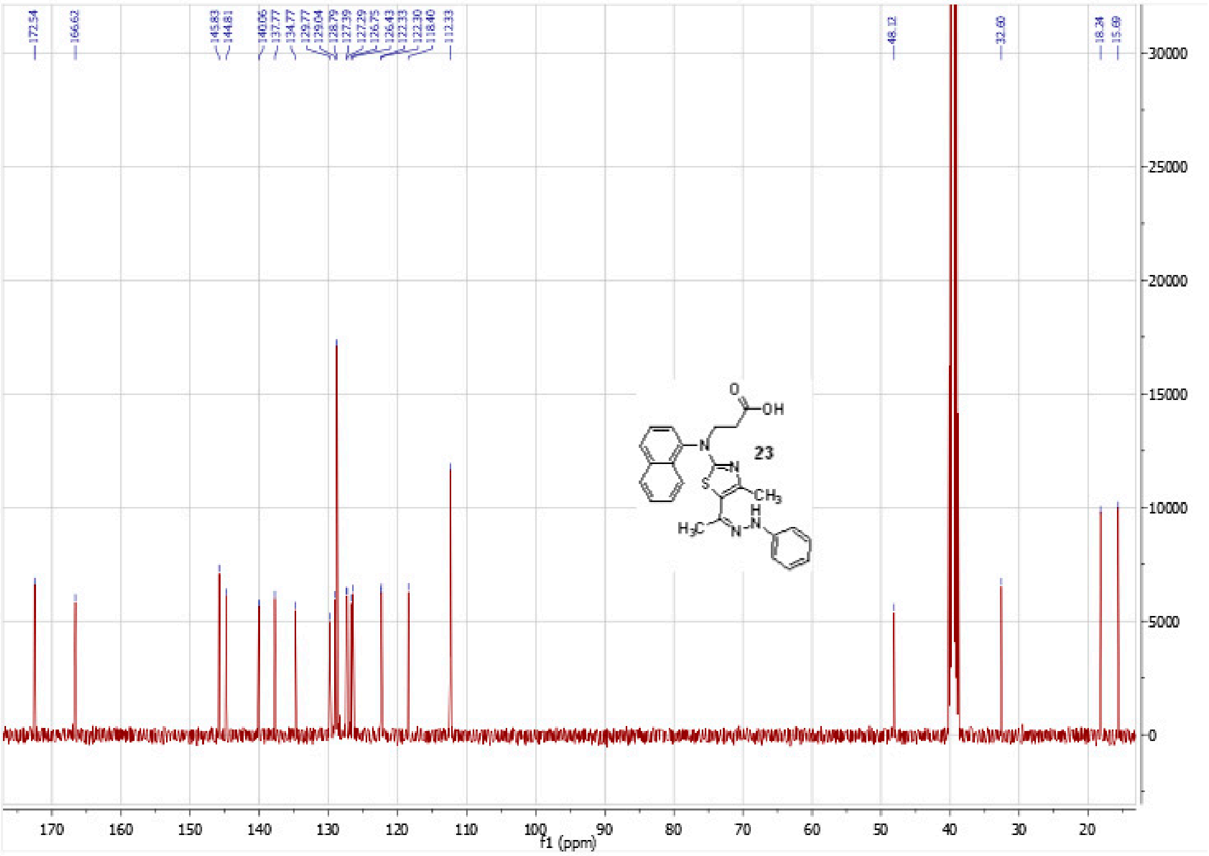

Figure S16: ^{13}C-NMR spectrum of compound **23**.

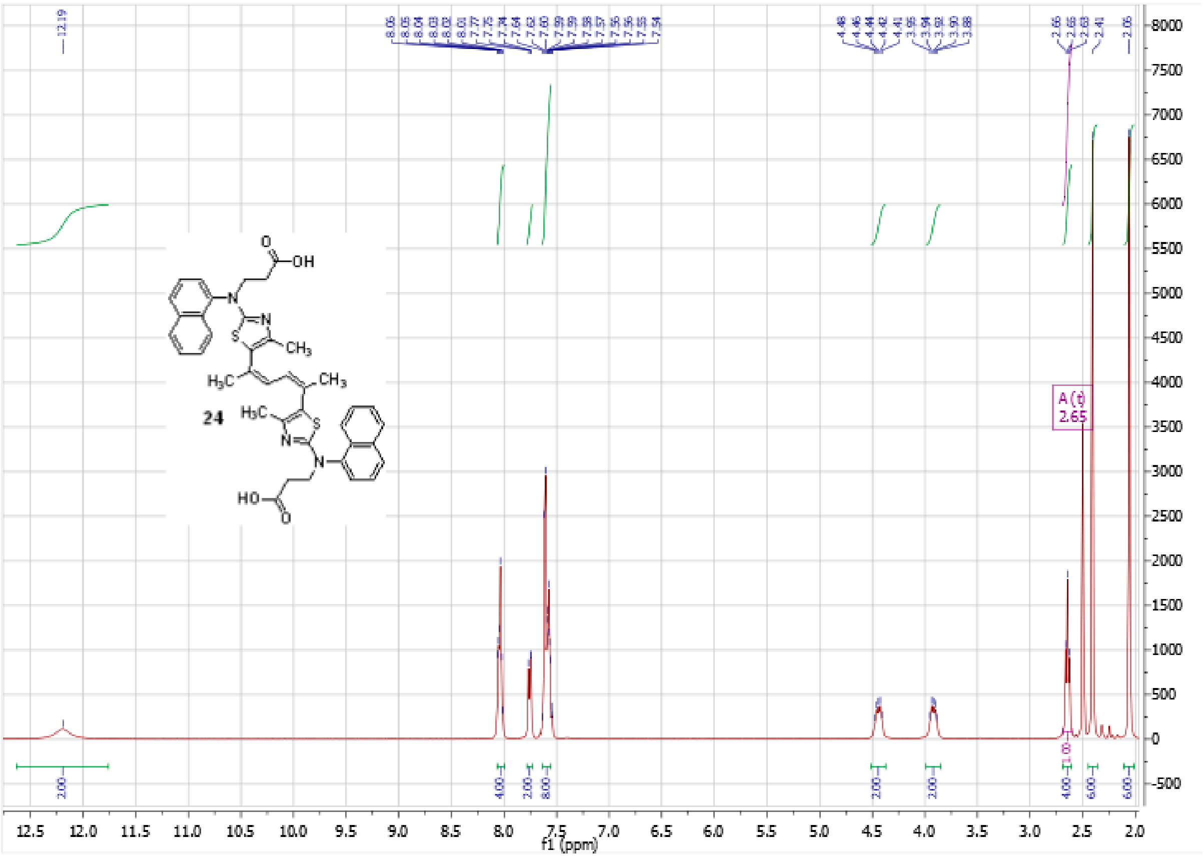

Figure S17: ^{1}H-NMR spectrum of compound **24**.

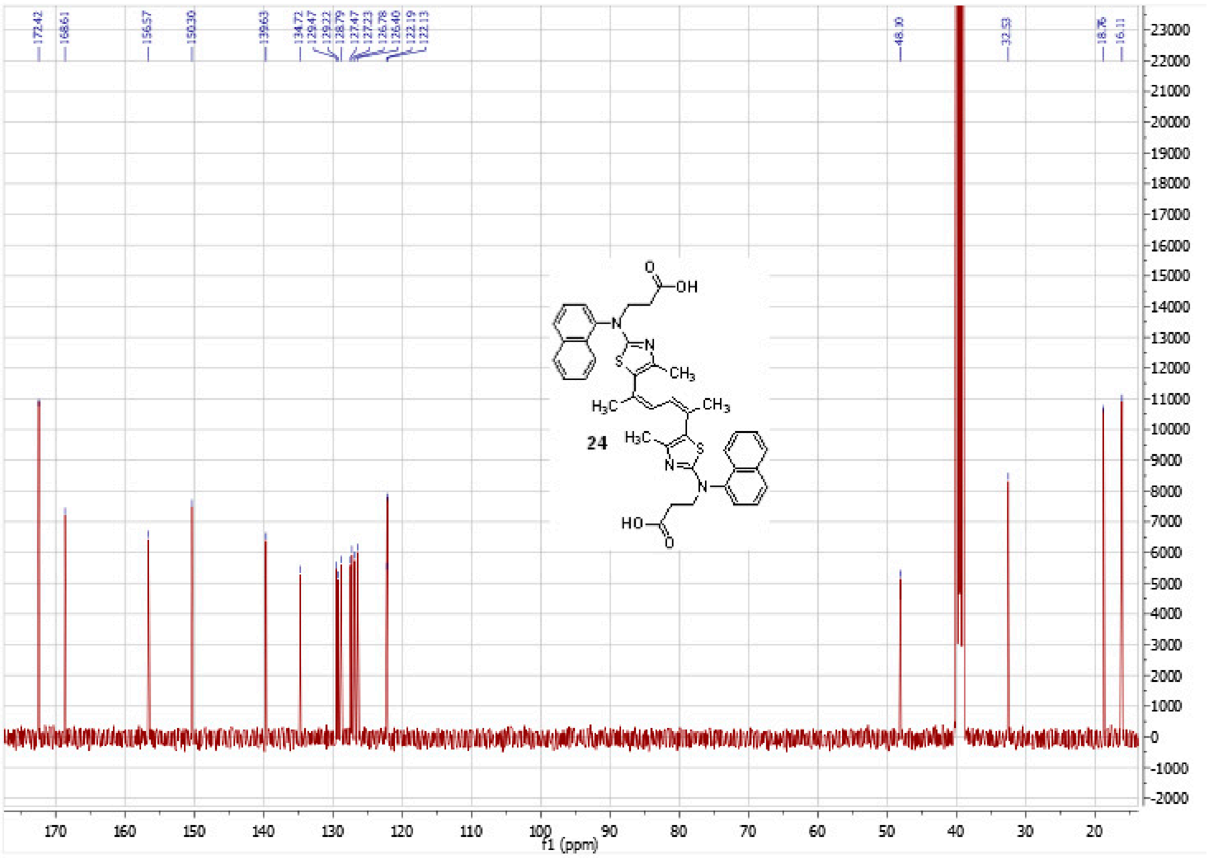

Figure S18: ^{13}C-NMR spectrum of compound **24**.

3. Dose-response curves of PL^{pro}

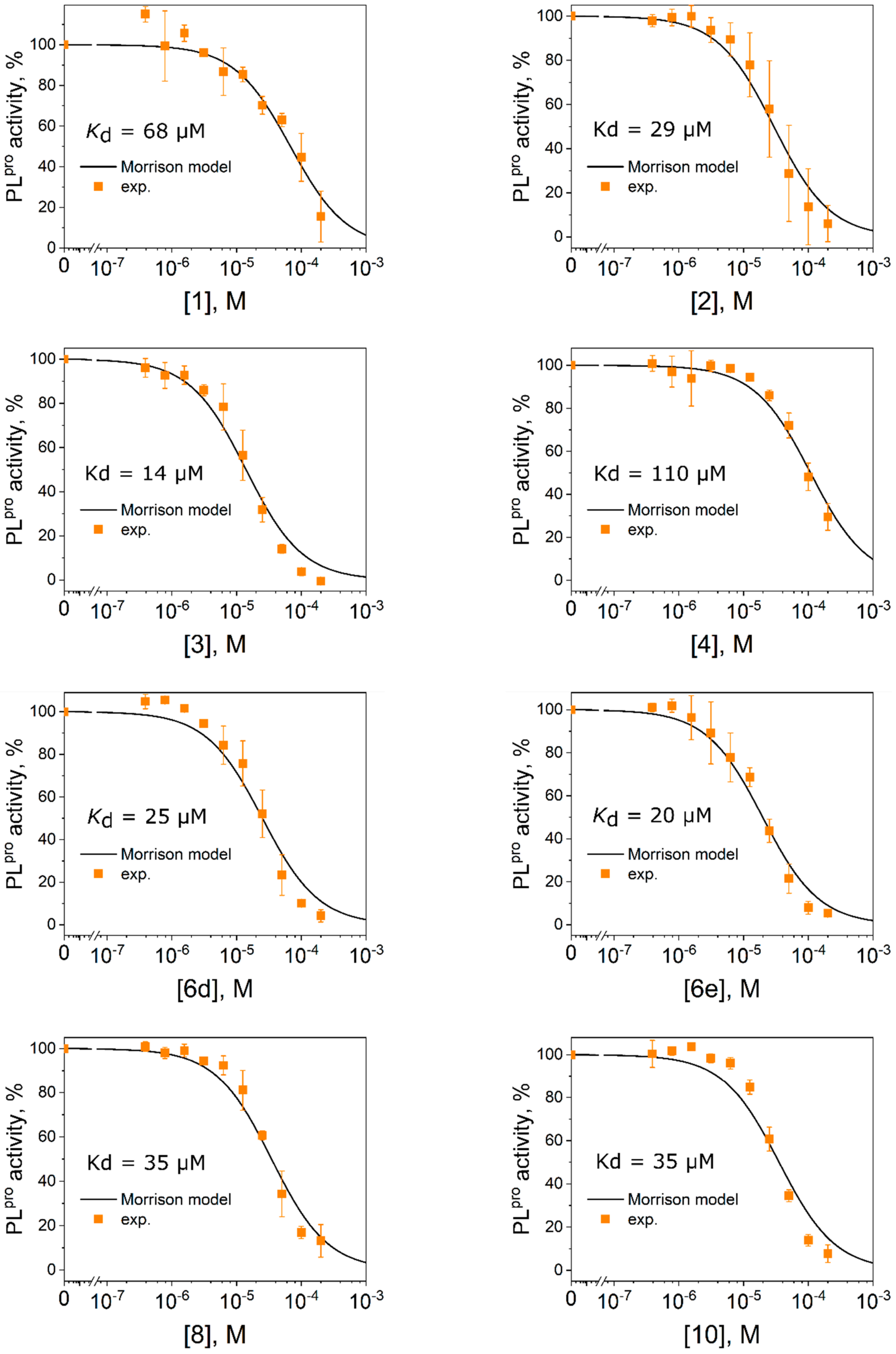

PL^{pro} activity, %
K_d = 68 μM
Morrison model
exp.
[1], M
Kd = 29 μM
[2], M
Kd = 14 μM
[3], M
Kd = 110 μM
[4], M
K_d = 25 μM
[6d], M
K_d = 20 μM
[6e], M
Kd = 35 μM
[8], M
Kd = 35 μM
[10], M
0
10^{-7}
10^{-6}
10^{-5}
10^{-4}
10^{-3}
0
20
40
60
80
100

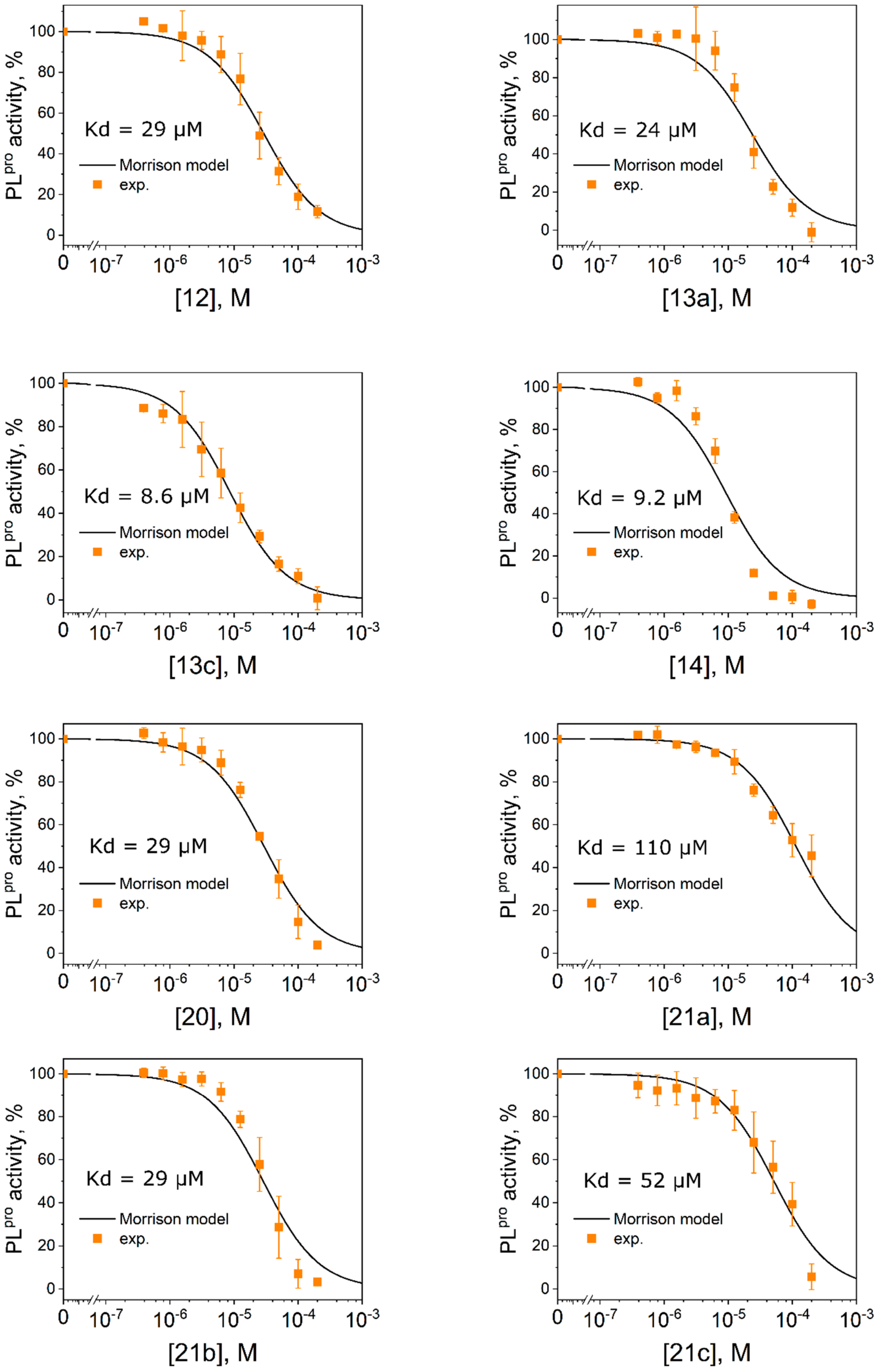
PL^{pro} activity, %
Kd = 29 μM
Morrison model
exp.
[12], M
Kd = 24 μM
[13a], M
Kd = 8.6 μM
[13c], M
Kd = 9.2 μM
[14], M
Kd = 29 μM
[20], M
Kd = 110 μM
[21a], M
Kd = 29 μM
[21b], M
Kd = 52 μM
[21c], M
0
10^{-7}
10^{-6}
10^{-5}
10^{-4}
10^{-3}

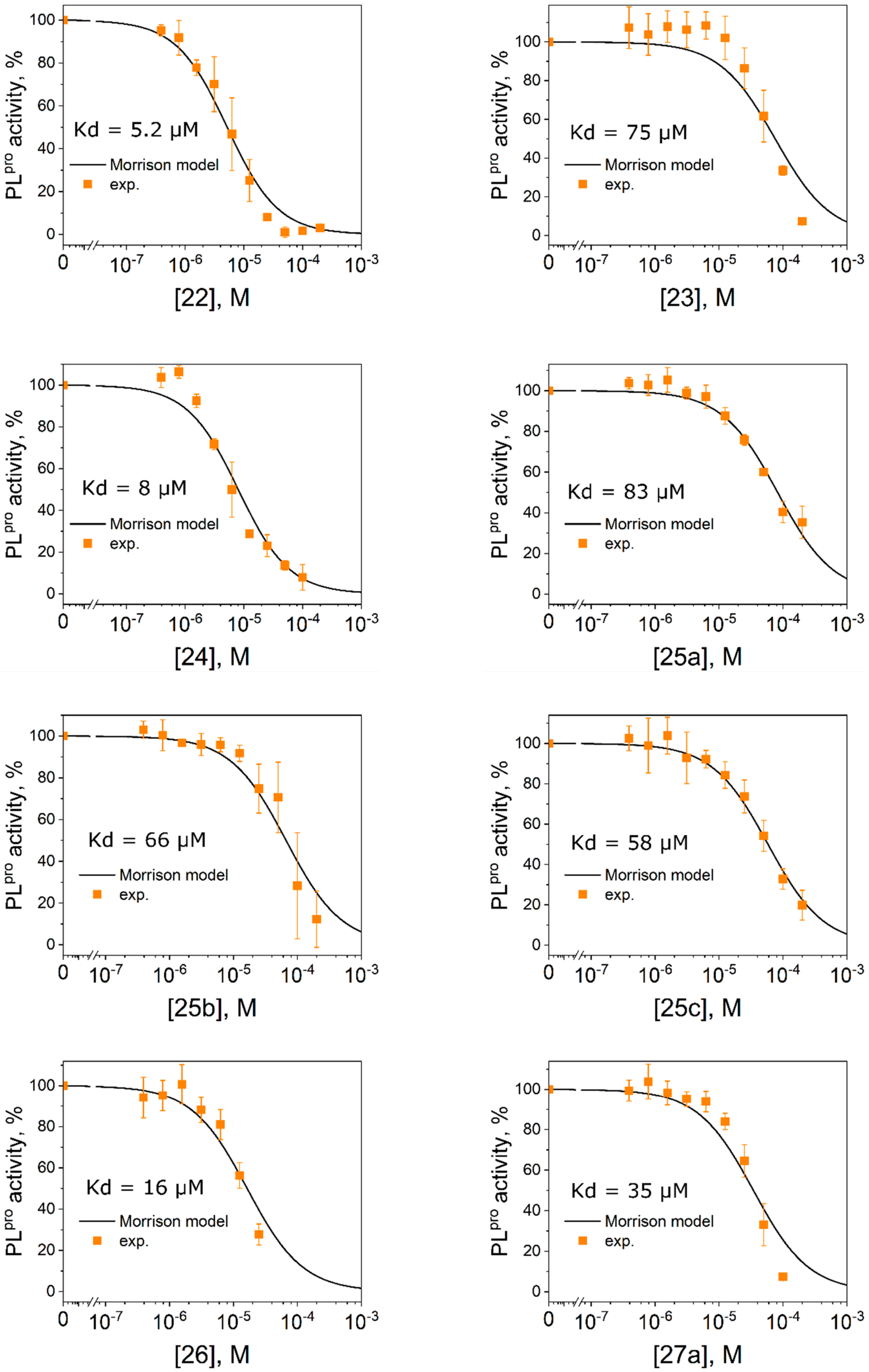

PLpro activity, %
Kd = 5.2 μM
Morrison model
exp.
[22], M
Kd = 75 μM
[23], M
Kd = 8 μM
[24], M
Kd = 83 μM
[25a], M
Kd = 66 μM
[25b], M
Kd = 58 μM
[25c], M
Kd = 16 μM
[26], M
Kd = 35 μM
[27a], M

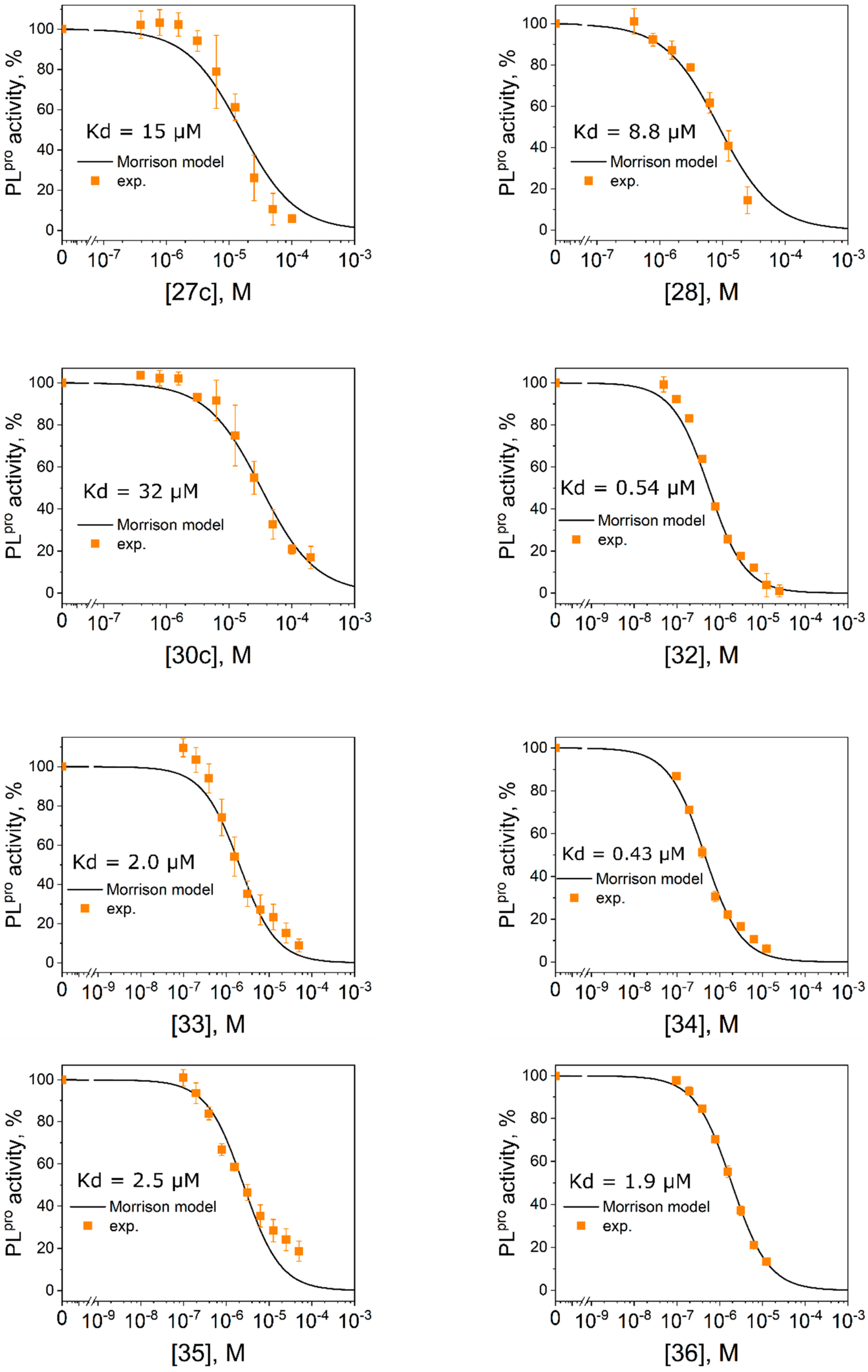

PLpro activity, %
Kd = 15 μM
Morrison model
exp.
[27c], M
Kd = 8.8 μM
Morrison model
exp.
[28], M
Kd = 32 μM
Morrison model
exp.
[30c], M
Kd = 0.54 μM
Morrison model
exp.
[32], M
Kd = 2.0 μM
Morrison model
exp.
[33], M
Kd = 0.43 μM
Morrison model
exp.
[34], M
Kd = 2.5 μM
Morrison model
exp.
[35], M
Kd = 1.9 μM
Morrison model
exp.
[36], M

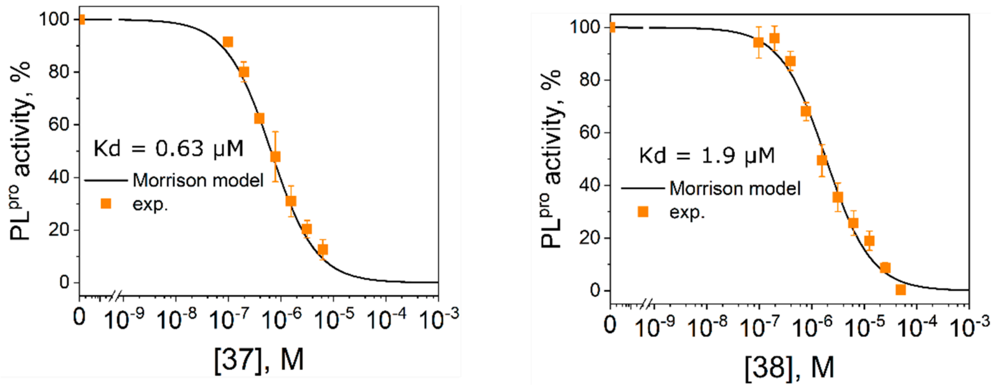

Figure S19. Dose-response curves of PL^{pro} inhibition with naphthalene-based and disulfide compounds.

4. Dose-response curves of M^{pro}.

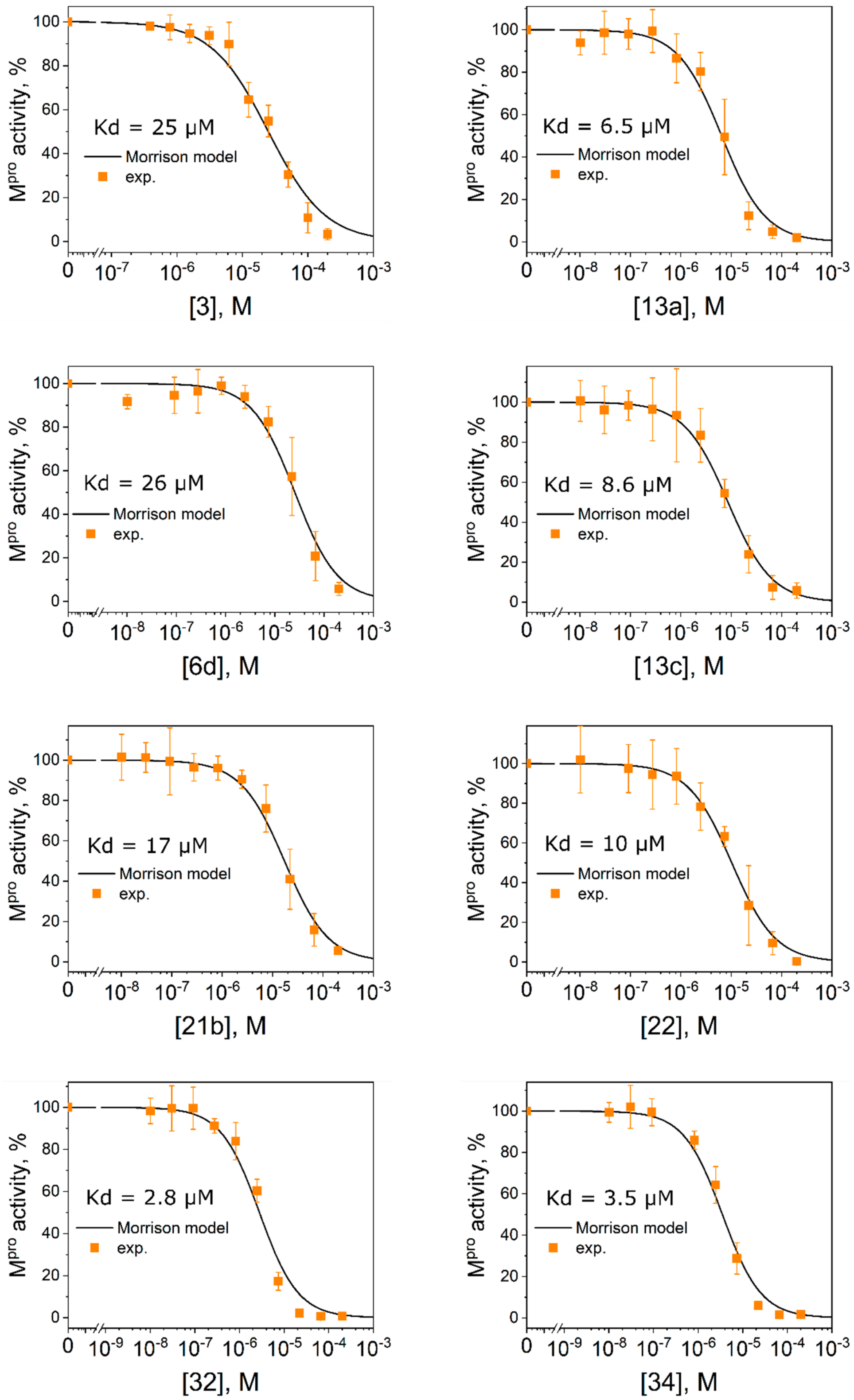

Figure S20. Dose-response curves of M^{pro} inhibition with naphthalene-based and disulfide compounds.

5. **Table S1**. Primers used in PL^{pro}C111S mutagenesis.

Primer name	Sequence
PL^{pro}-C111S_F	5' - gctgataacaattcctacctagctac - 3'
PL^{pro}-C111S_R	5' - ccatttgatgctagtcagacc - 3'

6. Sequencing analysis of PL^{pro} WT and PL^{pro}C111S plasmids.

The only one difference between two plasmids is nucleotide at 5468 position.

```
 1                                                      50
  PLpro_WT  TGGCGAATGG GACGCGCCCT GTAGCGGCGC ATTAAGCGCG GCGGGTGTGG
PLproC111S  TGGCGAATGG GACGCGCCCT GTAGCGGCGC ATTAAGCGCG GCGGGTGTGG
 Consensus  TGGCGAATGG GACGCGCCCT GTAGCGGCGC ATTAAGCGCG GCGGGTGTGG

            51                                                100
  PLpro_WT  TGGTTACGCG CAGCGTGACC GCTACACTTG CCAGCGCCCT AGCGCCCGCT
PLproC111S  TGGTTACGCG CAGCGTGACC GCTACACTTG CCAGCGCCCT AGCGCCCGCT
 Consensus  TGGTTACGCG CAGCGTGACC GCTACACTTG CCAGCGCCCT AGCGCCCGCT

            101                                               150
  PLpro_WT  CCTTTCGCTT TCTTCCCTTC CTTTCTCGCC ACGTTCGCCG GCTTTCCCCG
PLproC111S  CCTTTCGCTT TCTTCCCTTC CTTTCTCGCC ACGTTCGCCG GCTTTCCCCG
 Consensus  CCTTTCGCTT TCTTCCCTTC CTTTCTCGCC ACGTTCGCCG GCTTTCCCCG

            151                                               200
  PLpro_WT  TCAAGCTCTA AATCGGGGGC TCCCTTTAGG GTTCCGATTT AGTGCTTTAC
PLproC111S  TCAAGCTCTA AATCGGGGGC TCCCTTTAGG GTTCCGATTT AGTGCTTTAC
 Consensus  TCAAGCTCTA AATCGGGGGC TCCCTTTAGG GTTCCGATTT AGTGCTTTAC

            201                                               250
  PLpro_WT  GGCACCTCGA CCCCAAAAAA CTTGATTAGG GTGATGGTTC ACGTAGTGGG
PLproC111S  GGCACCTCGA CCCCAAAAAA CTTGATTAGG GTGATGGTTC ACGTAGTGGG
 Consensus  GGCACCTCGA CCCCAAAAAA CTTGATTAGG GTGATGGTTC ACGTAGTGGG

            251                                               300
  PLpro_WT  CCATCGCCCT GATAGACGGT TTTTCGCCCT TTGACGTTGG AGTCCACGTT
PLproC111S  CCATCGCCCT GATAGACGGT TTTTCGCCCT TTGACGTTGG AGTCCACGTT
 Consensus  CCATCGCCCT GATAGACGGT TTTTCGCCCT TTGACGTTGG AGTCCACGTT

            301                                               350
  PLpro_WT  CTTTAATAGT GGACTCTTGT TCCAAACTGG AACAACACTC AACCCTATCT
PLproC111S  CTTTAATAGT GGACTCTTGT TCCAAACTGG AACAACACTC AACCCTATCT
 Consensus  CTTTAATAGT GGACTCTTGT TCCAAACTGG AACAACACTC AACCCTATCT

            351                                               400
  PLpro_WT  CGGTCTATTC TTTTGATTTA TAAGGGATTT TGCCGATTTC GGCCTATTGG
PLproC111S  CGGTCTATTC TTTTGATTTA TAAGGGATTT TGCCGATTTC GGCCTATTGG
 Consensus  CGGTCTATTC TTTTGATTTA TAAGGGATTT TGCCGATTTC GGCCTATTGG

            401                                               450
  PLpro_WT  TTAAAAAATG AGCTGATTTA ACAAAAATTT AACGCGAATT TTAACAAAAT
PLproC111S  TTAAAAAATG AGCTGATTTA ACAAAAATTT AACGCGAATT TTAACAAAAT
```

```
 Consensus  TTAAAAAATG AGCTGATTTA ACAAAAATTT AACGCGAATT TTAACAAAAT

            451                                              500
  PLpro_WT  ATTAACGTTT ACAATTTCAG GTGGCACTTT TCGGGGAAAT GTGCGCGGAA
PLproC111S  ATTAACGTTT ACAATTTCAG GTGGCACTTT TCGGGGAAAT GTGCGCGGAA
 Consensus  ATTAACGTTT ACAATTTCAG GTGGCACTTT TCGGGGAAAT GTGCGCGGAA

            501                                              550
  PLpro_WT  CCCCTATTTG TTTATTTTTC TAAATACATT CAAATATGTA TCCGCTCATG
PLproC111S  CCCCTATTTG TTTATTTTTC TAAATACATT CAAATATGTA TCCGCTCATG
 Consensus  CCCCTATTTG TTTATTTTTC TAAATACATT CAAATATGTA TCCGCTCATG

            551                                              600
  PLpro_WT  AATTAATTCT TAGAAAAACT CATCGAGCAT CAAATGAAAC TGCAATTTAT
PLproC111S  AATTAATTCT TAGAAAAACT CATCGAGCAT CAAATGAAAC TGCAATTTAT
 Consensus  AATTAATTCT TAGAAAAACT CATCGAGCAT CAAATGAAAC TGCAATTTAT

            601                                              650
  PLpro_WT  TCATATCAGG ATTATCAATA CCATATTTTT GAAAAAGCCG TTTCTGTAAT
PLproC111S  TCATATCAGG ATTATCAATA CCATATTTTT GAAAAAGCCG TTTCTGTAAT
 Consensus  TCATATCAGG ATTATCAATA CCATATTTTT GAAAAAGCCG TTTCTGTAAT

            651                                              700
  PLpro_WT  GAAGGAGAAA ACTCACCGAG GCAGTTCCAT AGGATGGCAA GATCCTGGTA
PLproC111S  GAAGGAGAAA ACTCACCGAG GCAGTTCCAT AGGATGGCAA GATCCTGGTA
 Consensus  GAAGGAGAAA ACTCACCGAG GCAGTTCCAT AGGATGGCAA GATCCTGGTA

            701                                              750
  PLpro_WT  TCGGTCTGCG ATTCCGACTC GTCCAACATC AATACAACCT ATTAATTTCC
PLproC111S  TCGGTCTGCG ATTCCGACTC GTCCAACATC AATACAACCT ATTAATTTCC
 Consensus  TCGGTCTGCG ATTCCGACTC GTCCAACATC AATACAACCT ATTAATTTCC

            751                                              800
  PLpro_WT  CCTCGTCAAA AATAAGGTTA TCAAGTGAGA AATCACCATG AGTGACGACT
PLproC111S  CCTCGTCAAA AATAAGGTTA TCAAGTGAGA AATCACCATG AGTGACGACT
 Consensus  CCTCGTCAAA AATAAGGTTA TCAAGTGAGA AATCACCATG AGTGACGACT

            801                                              850
  PLpro_WT  GAATCCGGTG AGAATGGCAA AAGTTTATGC ATTTCTTTCC AGACTTGTTC
PLproC111S  GAATCCGGTG AGAATGGCAA AAGTTTATGC ATTTCTTTCC AGACTTGTTC
 Consensus  GAATCCGGTG AGAATGGCAA AAGTTTATGC ATTTCTTTCC AGACTTGTTC

            851                                              900
  PLpro_WT  AACAGGCCAG CCATTACGCT CGTCATCAAA ATCACTCGCA TCAACCAAAC
PLproC111S  AACAGGCCAG CCATTACGCT CGTCATCAAA ATCACTCGCA TCAACCAAAC
 Consensus  AACAGGCCAG CCATTACGCT CGTCATCAAA ATCACTCGCA TCAACCAAAC

            901                                              950
  PLpro_WT  CGTTATTCAT TCGTGATTGC GCCTGAGCGA GACGAAATAC GCGATCGCTG
PLproC111S  CGTTATTCAT TCGTGATTGC GCCTGAGCGA GACGAAATAC GCGATCGCTG
 Consensus  CGTTATTCAT TCGTGATTGC GCCTGAGCGA GACGAAATAC GCGATCGCTG
```

```
             951                                                1000
  PLpro_WT   TTAAAAGGAC AATTACAAAC AGGAATCGAA TGCAACCGGC GCAGGAACAC
PLproC111S   TTAAAAGGAC AATTACAAAC AGGAATCGAA TGCAACCGGC GCAGGAACAC
 Consensus   TTAAAAGGAC AATTACAAAC AGGAATCGAA TGCAACCGGC GCAGGAACAC

             1001                                               1050
  PLpro_WT   TGCCAGCGCA TCAACAATAT TTTCACCTGA ATCAGGATAT TCTTCTAATA
PLproC111S   TGCCAGCGCA TCAACAATAT TTTCACCTGA ATCAGGATAT TCTTCTAATA
 Consensus   TGCCAGCGCA TCAACAATAT TTTCACCTGA ATCAGGATAT TCTTCTAATA

             1051                                               1100
  PLpro_WT   CCTGGAATGC TGTTTTCCCG GGGATCGCAG TGGTGAGTAA CCATGCATCA
PLproC111S   CCTGGAATGC TGTTTTCCCG GGGATCGCAG TGGTGAGTAA CCATGCATCA
 Consensus   CCTGGAATGC TGTTTTCCCG GGGATCGCAG TGGTGAGTAA CCATGCATCA

             1101                                               1150
  PLpro_WT   TCAGGAGTAC GGATAAAATG CTTGATGGTC GGAAGAGGCA TAAATTCCGT
PLproC111S   TCAGGAGTAC GGATAAAATG CTTGATGGTC GGAAGAGGCA TAAATTCCGT
 Consensus   TCAGGAGTAC GGATAAAATG CTTGATGGTC GGAAGAGGCA TAAATTCCGT

             1151                                               1200
  PLpro_WT   CAGCCAGTTT AGTCTGACCA TCTCATCTGT AACATCATTG GCAACGCTAC
PLproC111S   CAGCCAGTTT AGTCTGACCA TCTCATCTGT AACATCATTG GCAACGCTAC
 Consensus   CAGCCAGTTT AGTCTGACCA TCTCATCTGT AACATCATTG GCAACGCTAC

             1201                                               1250
  PLpro_WT   CTTTGCCATG TTTCAGAAAC AACTCTGGCG CATCGGGCTT CCCATACAAT
PLproC111S   CTTTGCCATG TTTCAGAAAC AACTCTGGCG CATCGGGCTT CCCATACAAT
 Consensus   CTTTGCCATG TTTCAGAAAC AACTCTGGCG CATCGGGCTT CCCATACAAT

             1251                                               1300
  PLpro_WT   CGATAGATTG TCGCACCTGA TTGCCCGACA TTATCGCGAG CCCATTTATA
PLproC111S   CGATAGATTG TCGCACCTGA TTGCCCGACA TTATCGCGAG CCCATTTATA
 Consensus   CGATAGATTG TCGCACCTGA TTGCCCGACA TTATCGCGAG CCCATTTATA

             1301                                               1350
  PLpro_WT   CCCATATAAA TCAGCATCCA TGTTGGAATT TAATCGCGGC CTAGAGCAAG
PLproC111S   CCCATATAAA TCAGCATCCA TGTTGGAATT TAATCGCGGC CTAGAGCAAG
 Consensus   CCCATATAAA TCAGCATCCA TGTTGGAATT TAATCGCGGC CTAGAGCAAG

             1351                                               1400
  PLpro_WT   ACGTTTCCCG TTGAATATGG CTCATAACAC CCCTTGTATT ACTGTTTATG
PLproC111S   ACGTTTCCCG TTGAATATGG CTCATAACAC CCCTTGTATT ACTGTTTATG
 Consensus   ACGTTTCCCG TTGAATATGG CTCATAACAC CCCTTGTATT ACTGTTTATG

             1401                                               1450
  PLpro_WT   TAAGCAGACA GTTTTATTGT TCATGACCAA AATCCCTTAA CGTGAGTTTT
PLproC111S   TAAGCAGACA GTTTTATTGT TCATGACCAA AATCCCTTAA CGTGAGTTTT
 Consensus   TAAGCAGACA GTTTTATTGT TCATGACCAA AATCCCTTAA CGTGAGTTTT

             1451                                               1500
  PLpro_WT   CGTTCCACTG AGCGTCAGAC CCCGTAGAAA AGATCAAAGG ATCTTCTTGA
```

```
PLproC111S  CGTTCCACTG AGCGTCAGAC CCCGTAGAAA AGATCAAAGG ATCTTCTTGA
 Consensus  CGTTCCACTG AGCGTCAGAC CCCGTAGAAA AGATCAAAGG ATCTTCTTGA

            1501                                              1550
  PLpro_WT  GATCCTTTTT TTCTGCGCGT AATCTGCTGC TTGCAAACAA AAAAACCACC
PLproC111S  GATCCTTTTT TTCTGCGCGT AATCTGCTGC TTGCAAACAA AAAAACCACC
 Consensus  GATCCTTTTT TTCTGCGCGT AATCTGCTGC TTGCAAACAA AAAAACCACC

            1551                                              1600
  PLpro_WT  GCTACCAGCG GTGGTTTGTT TGCCGGATCA AGAGCTACCA ACTCTTTTTC
PLproC111S  GCTACCAGCG GTGGTTTGTT TGCCGGATCA AGAGCTACCA ACTCTTTTTC
 Consensus  GCTACCAGCG GTGGTTTGTT TGCCGGATCA AGAGCTACCA ACTCTTTTTC

            1601                                              1650
  PLpro_WT  CGAAGGTAAC TGGCTTCAGC AGAGCGCAGA TACCAAATAC TGTCCTTCTA
PLproC111S  CGAAGGTAAC TGGCTTCAGC AGAGCGCAGA TACCAAATAC TGTCCTTCTA
 Consensus  CGAAGGTAAC TGGCTTCAGC AGAGCGCAGA TACCAAATAC TGTCCTTCTA

            1651                                              1700
  PLpro_WT  GTGTAGCCGT AGTTAGGCCA CCACTTCAAG AACTCTGTAG CACCGCCTAC
PLproC111S  GTGTAGCCGT AGTTAGGCCA CCACTTCAAG AACTCTGTAG CACCGCCTAC
 Consensus  GTGTAGCCGT AGTTAGGCCA CCACTTCAAG AACTCTGTAG CACCGCCTAC

            1701                                              1750
  PLpro_WT  ATACCTCGCT CTGCTAATCC TGTTACCAGT GGCTGCTGCC AGTGGCGATA
PLproC111S  ATACCTCGCT CTGCTAATCC TGTTACCAGT GGCTGCTGCC AGTGGCGATA
 Consensus  ATACCTCGCT CTGCTAATCC TGTTACCAGT GGCTGCTGCC AGTGGCGATA

            1751                                              1800
  PLpro_WT  AGTCGTGTCT TACCGGGTTG GACTCAAGAC GATAGTTACC GGATAAGGCG
PLproC111S  AGTCGTGTCT TACCGGGTTG GACTCAAGAC GATAGTTACC GGATAAGGCG
 Consensus  AGTCGTGTCT TACCGGGTTG GACTCAAGAC GATAGTTACC GGATAAGGCG

            1801                                              1850
  PLpro_WT  CAGCGGTCGG GCTGAACGGG GGGTTCGTGC ACACAGCCCA GCTTGGAGCG
PLproC111S  CAGCGGTCGG GCTGAACGGG GGGTTCGTGC ACACAGCCCA GCTTGGAGCG
 Consensus  CAGCGGTCGG GCTGAACGGG GGGTTCGTGC ACACAGCCCA GCTTGGAGCG

            1851                                              1900
  PLpro_WT  AACGACCTAC ACCGAACTGA GATACCTACA GCGTGAGCTA TGAGAAAGCG
PLproC111S  AACGACCTAC ACCGAACTGA GATACCTACA GCGTGAGCTA TGAGAAAGCG
 Consensus  AACGACCTAC ACCGAACTGA GATACCTACA GCGTGAGCTA TGAGAAAGCG

            1901                                              1950
  PLpro_WT  CCACGCTTCC CGAAGGGAGA AAGGCGGACA GGTATCCGGT AAGCGGCAGG
PLproC111S  CCACGCTTCC CGAAGGGAGA AAGGCGGACA GGTATCCGGT AAGCGGCAGG
 Consensus  CCACGCTTCC CGAAGGGAGA AAGGCGGACA GGTATCCGGT AAGCGGCAGG

            1951                                              2000
  PLpro_WT  GTCGGAACAG GAGAGCGCAC GAGGGAGCTT CCAGGGGGAA ACGCCTGGTA
PLproC111S  GTCGGAACAG GAGAGCGCAC GAGGGAGCTT CCAGGGGGAA ACGCCTGGTA
 Consensus  GTCGGAACAG GAGAGCGCAC GAGGGAGCTT CCAGGGGGAA ACGCCTGGTA
```

```
             2001                                               2050
  PLpro_WT   TCTTTATAGT CCTGTCGGGT TTCGCCACCT CTGACTTGAG CGTCGATTTT
PLproC111S   TCTTTATAGT CCTGTCGGGT TTCGCCACCT CTGACTTGAG CGTCGATTTT
 Consensus   TCTTTATAGT CCTGTCGGGT TTCGCCACCT CTGACTTGAG CGTCGATTTT

             2051                                               2100
  PLpro_WT   TGTGATGCTC GTCAGGGGGG CGGAGCCTAT GGAAAAACGC CAGCAACGCG
PLproC111S   TGTGATGCTC GTCAGGGGGG CGGAGCCTAT GGAAAAACGC CAGCAACGCG
 Consensus   TGTGATGCTC GTCAGGGGGG CGGAGCCTAT GGAAAAACGC CAGCAACGCG

             2101                                               2150
  PLpro_WT   GCCTTTTTAC GGTTCCTGGC CTTTTGCTGG CCTTTTGCTC ACATGTTCTT
PLproC111S   GCCTTTTTAC GGTTCCTGGC CTTTTGCTGG CCTTTTGCTC ACATGTTCTT
 Consensus   GCCTTTTTAC GGTTCCTGGC CTTTTGCTGG CCTTTTGCTC ACATGTTCTT

             2151                                               2200
  PLpro_WT   TCCTGCGTTA TCCCCTGATT CTGTGGATAA CCGTATTACC GCCTTTGAGT
PLproC111S   TCCTGCGTTA TCCCCTGATT CTGTGGATAA CCGTATTACC GCCTTTGAGT
 Consensus   TCCTGCGTTA TCCCCTGATT CTGTGGATAA CCGTATTACC GCCTTTGAGT

             2201                                               2250
  PLpro_WT   GAGCTGATAC CGCTCGCCGC AGCCGAACGA CCGAGCGCAG CGAGTCAGTG
PLproC111S   GAGCTGATAC CGCTCGCCGC AGCCGAACGA CCGAGCGCAG CGAGTCAGTG
 Consensus   GAGCTGATAC CGCTCGCCGC AGCCGAACGA CCGAGCGCAG CGAGTCAGTG

             2251                                               2300
  PLpro_WT   AGCGAGGAAG CGGAAGAGCG CCTGATGCGG TATTTTCTCC TTACGCATCT
PLproC111S   AGCGAGGAAG CGGAAGAGCG CCTGATGCGG TATTTTCTCC TTACGCATCT
 Consensus   AGCGAGGAAG CGGAAGAGCG CCTGATGCGG TATTTTCTCC TTACGCATCT

             2301                                               2350
  PLpro_WT   GTGCGGTATT TCACACCGCA TATATGGTGC ACTCTCAGTA CAATCTGCTC
PLproC111S   GTGCGGTATT TCACACCGCA TATATGGTGC ACTCTCAGTA CAATCTGCTC
 Consensus   GTGCGGTATT TCACACCGCA TATATGGTGC ACTCTCAGTA CAATCTGCTC

             2351                                               2400
  PLpro_WT   TGATGCCGCA TAGTTAAGCC AGTATACACT CCGCTATCGC TACGTGACTG
PLproC111S   TGATGCCGCA TAGTTAAGCC AGTATACACT CCGCTATCGC TACGTGACTG
 Consensus   TGATGCCGCA TAGTTAAGCC AGTATACACT CCGCTATCGC TACGTGACTG

             2401                                               2450
  PLpro_WT   GGTCATGGCT GCGCCCCGAC ACCCGCCAAC ACCCGCTGAC GCGCCCTGAC
PLproC111S   GGTCATGGCT GCGCCCCGAC ACCCGCCAAC ACCCGCTGAC GCGCCCTGAC
 Consensus   GGTCATGGCT GCGCCCCGAC ACCCGCCAAC ACCCGCTGAC GCGCCCTGAC

             2451                                               2500
  PLpro_WT   GGGCTTGTCT GCTCCCGGCA TCCGCTTACA GACAAGCTGT GACCGTCTCC
PLproC111S   GGGCTTGTCT GCTCCCGGCA TCCGCTTACA GACAAGCTGT GACCGTCTCC
 Consensus   GGGCTTGTCT GCTCCCGGCA TCCGCTTACA GACAAGCTGT GACCGTCTCC

             2501                                               2550
```

```
  PLpro_WT  GGGAGCTGCA TGTGTCAGAG GTTTTCACCG TCATCACCGA AACGCGCGAG
PLproC111S  GGGAGCTGCA TGTGTCAGAG GTTTTCACCG TCATCACCGA AACGCGCGAG
 Consensus  GGGAGCTGCA TGTGTCAGAG GTTTTCACCG TCATCACCGA AACGCGCGAG

            2551                                              2600
  PLpro_WT  GCAGCTGCGG TAAAGCTCAT CAGCGTGGTC GTGAAGCGAT TCACAGATGT
PLproC111S  GCAGCTGCGG TAAAGCTCAT CAGCGTGGTC GTGAAGCGAT TCACAGATGT
 Consensus  GCAGCTGCGG TAAAGCTCAT CAGCGTGGTC GTGAAGCGAT TCACAGATGT

            2601                                              2650
  PLpro_WT  CTGCCTGTTC ATCCGCGTCC AGCTCGTTGA GTTTCTCCAG AAGCGTTAAT
PLproC111S  CTGCCTGTTC ATCCGCGTCC AGCTCGTTGA GTTTCTCCAG AAGCGTTAAT
 Consensus  CTGCCTGTTC ATCCGCGTCC AGCTCGTTGA GTTTCTCCAG AAGCGTTAAT

            2651                                              2700
  PLpro_WT  GTCTGGCTTC TGATAAAGCG GGCCATGTTA AGGGCGGTTT TTTCCTGTTT
PLproC111S  GTCTGGCTTC TGATAAAGCG GGCCATGTTA AGGGCGGTTT TTTCCTGTTT
 Consensus  GTCTGGCTTC TGATAAAGCG GGCCATGTTA AGGGCGGTTT TTTCCTGTTT

            2701                                              2750
  PLpro_WT  GGTCACTGAT GCCTCCGTGT AAGGGGGATT TCTGTTCATG GGGGTAATGA
PLproC111S  GGTCACTGAT GCCTCCGTGT AAGGGGGATT TCTGTTCATG GGGGTAATGA
 Consensus  GGTCACTGAT GCCTCCGTGT AAGGGGGATT TCTGTTCATG GGGGTAATGA

            2751                                              2800
  PLpro_WT  TACCGATGAA ACGAGAGAGG ATGCTCACGA TACGGGTTAC TGATGATGAA
PLproC111S  TACCGATGAA ACGAGAGAGG ATGCTCACGA TACGGGTTAC TGATGATGAA
 Consensus  TACCGATGAA ACGAGAGAGG ATGCTCACGA TACGGGTTAC TGATGATGAA

            2801                                              2850
  PLpro_WT  CATGCCCGGT TACTGGAACG TTGTGAGGGT AAACAACTGG CGGTATGGAT
PLproC111S  CATGCCCGGT TACTGGAACG TTGTGAGGGT AAACAACTGG CGGTATGGAT
 Consensus  CATGCCCGGT TACTGGAACG TTGTGAGGGT AAACAACTGG CGGTATGGAT

            2851                                              2900
  PLpro_WT  GCGGCGGGAC CAGAGAAAAA TCACTCAGGG TCAATGCCAG CGCTTCGTTA
PLproC111S  GCGGCGGGAC CAGAGAAAAA TCACTCAGGG TCAATGCCAG CGCTTCGTTA
 Consensus  GCGGCGGGAC CAGAGAAAAA TCACTCAGGG TCAATGCCAG CGCTTCGTTA

            2901                                              2950
  PLpro_WT  ATACAGATGT AGGTGTTCCA CAGGGTAGCC AGCAGCATCC TGCGATGCAG
PLproC111S  ATACAGATGT AGGTGTTCCA CAGGGTAGCC AGCAGCATCC TGCGATGCAG
 Consensus  ATACAGATGT AGGTGTTCCA CAGGGTAGCC AGCAGCATCC TGCGATGCAG

            2951                                              3000
  PLpro_WT  ATCCGGAACA TAATGGTGCA GGGCGCTGAC TTCCGCGTTT CCAGACTTTA
PLproC111S  ATCCGGAACA TAATGGTGCA GGGCGCTGAC TTCCGCGTTT CCAGACTTTA
 Consensus  ATCCGGAACA TAATGGTGCA GGGCGCTGAC TTCCGCGTTT CCAGACTTTA

            3001                                              3050
  PLpro_WT  CGAAACACGG AAACCGAAGA CCATTCATGT TGTTGCTCAG GTCGCAGACG
PLproC111S  CGAAACACGG AAACCGAAGA CCATTCATGT TGTTGCTCAG GTCGCAGACG
```

```
Consensus   CGAAACACGG AAACCGAAGA CCATTCATGT TGTTGCTCAG GTCGCAGACG

            3051                                              3100
PLpro_WT    TTTTGCAGCA GCAGTCGCTT CACGTTCGCT CGCGTATCGG TGATTCATTC
PLproC111S  TTTTGCAGCA GCAGTCGCTT CACGTTCGCT CGCGTATCGG TGATTCATTC
Consensus   TTTTGCAGCA GCAGTCGCTT CACGTTCGCT CGCGTATCGG TGATTCATTC

            3101                                              3150
PLpro_WT    TGCTAACCAG TAAGGCAACC CCGCCAGCCT AGCCGGGTCC TCAACGACAG
PLproC111S  TGCTAACCAG TAAGGCAACC CCGCCAGCCT AGCCGGGTCC TCAACGACAG
Consensus   TGCTAACCAG TAAGGCAACC CCGCCAGCCT AGCCGGGTCC TCAACGACAG

            3151                                              3200
PLpro_WT    GAGCACGATC ATGCGCACCC GTGGGGCCGC CATGCCGGCG ATAATGGCCT
PLproC111S  GAGCACGATC ATGCGCACCC GTGGGGCCGC CATGCCGGCG ATAATGGCCT
Consensus   GAGCACGATC ATGCGCACCC GTGGGGCCGC CATGCCGGCG ATAATGGCCT

            3201                                              3250
PLpro_WT    GCTTCTCGCC GAAACGTTTG GTGGCGGGAC CAGTGACGAA GGCTTGAGCG
PLproC111S  GCTTCTCGCC GAAACGTTTG GTGGCGGGAC CAGTGACGAA GGCTTGAGCG
Consensus   GCTTCTCGCC GAAACGTTTG GTGGCGGGAC CAGTGACGAA GGCTTGAGCG

            3251                                              3300
PLpro_WT    AGGGCGTGCA AGATTCCGAA TACCGCAAGC GACAGGCCGA TCATCGTCGC
PLproC111S  AGGGCGTGCA AGATTCCGAA TACCGCAAGC GACAGGCCGA TCATCGTCGC
Consensus   AGGGCGTGCA AGATTCCGAA TACCGCAAGC GACAGGCCGA TCATCGTCGC

            3301                                              3350
PLpro_WT    GCTCCAGCGA AAGCGGTCCT CGCCGAAAAT GACCCAGAGC GCTGCCGGCA
PLproC111S  GCTCCAGCGA AAGCGGTCCT CGCCGAAAAT GACCCAGAGC GCTGCCGGCA
Consensus   GCTCCAGCGA AAGCGGTCCT CGCCGAAAAT GACCCAGAGC GCTGCCGGCA

            3351                                              3400
PLpro_WT    CCTGTCCTAC GAGTTGCATG ATAAAGAAGA CAGTCATAAG TGCGGCGACG
PLproC111S  CCTGTCCTAC GAGTTGCATG ATAAAGAAGA CAGTCATAAG TGCGGCGACG
Consensus   CCTGTCCTAC GAGTTGCATG ATAAAGAAGA CAGTCATAAG TGCGGCGACG

            3401                                              3450
PLpro_WT    ATAGTCATGC CCCGCGCCCA CCGGAAGGAG CTGACTGGGT TGAAGGCTCT
PLproC111S  ATAGTCATGC CCCGCGCCCA CCGGAAGGAG CTGACTGGGT TGAAGGCTCT
Consensus   ATAGTCATGC CCCGCGCCCA CCGGAAGGAG CTGACTGGGT TGAAGGCTCT

            3451                                              3500
PLpro_WT    CAAGGGCATC GGTCGAGATC CCGGTGCCTA ATGAGTGAGC TAACTTACAT
PLproC111S  CAAGGGCATC GGTCGAGATC CCGGTGCCTA ATGAGTGAGC TAACTTACAT
Consensus   CAAGGGCATC GGTCGAGATC CCGGTGCCTA ATGAGTGAGC TAACTTACAT

            3501                                              3550
PLpro_WT    TAATTGCGTT GCGCTCACTG CCCGCTTTCC AGTCGGGAAA CCTGTCGTGC
PLproC111S  TAATTGCGTT GCGCTCACTG CCCGCTTTCC AGTCGGGAAA CCTGTCGTGC
Consensus   TAATTGCGTT GCGCTCACTG CCCGCTTTCC AGTCGGGAAA CCTGTCGTGC
```

```
           3551                                              3600
  PLpro_WT  CAGCTGCATT AATGAATCGG CCAACGCGCG GGGAGAGGCG GTTTGCGTAT
PLproC111S  CAGCTGCATT AATGAATCGG CCAACGCGCG GGGAGAGGCG GTTTGCGTAT
 Consensus  CAGCTGCATT AATGAATCGG CCAACGCGCG GGGAGAGGCG GTTTGCGTAT

           3601                                              3650
  PLpro_WT  TGGGCGCCAG GGTGGTTTTT CTTTTCACCA GTGAGACGGG CAACAGCTGA
PLproC111S  TGGGCGCCAG GGTGGTTTTT CTTTTCACCA GTGAGACGGG CAACAGCTGA
 Consensus  TGGGCGCCAG GGTGGTTTTT CTTTTCACCA GTGAGACGGG CAACAGCTGA

           3651                                              3700
  PLpro_WT  TTGCCCTTCA CCGCCTGGCC CTGAGAGAGT TGCAGCAAGC GGTCCACGCT
PLproC111S  TTGCCCTTCA CCGCCTGGCC CTGAGAGAGT TGCAGCAAGC GGTCCACGCT
 Consensus  TTGCCCTTCA CCGCCTGGCC CTGAGAGAGT TGCAGCAAGC GGTCCACGCT

           3701                                              3750
  PLpro_WT  GGTTTGCCCC AGCAGGCGAA AATCCTGTTT GATGGTGGTT AACGGCGGGA
PLproC111S  GGTTTGCCCC AGCAGGCGAA AATCCTGTTT GATGGTGGTT AACGGCGGGA
 Consensus  GGTTTGCCCC AGCAGGCGAA AATCCTGTTT GATGGTGGTT AACGGCGGGA

           3751                                              3800
  PLpro_WT  TATAACATGA GCTGTCTTCG GTATCGTCGT ATCCCACTAC CGAGATATCC
PLproC111S  TATAACATGA GCTGTCTTCG GTATCGTCGT ATCCCACTAC CGAGATATCC
 Consensus  TATAACATGA GCTGTCTTCG GTATCGTCGT ATCCCACTAC CGAGATATCC

           3801                                              3850
  PLpro_WT  GCACCAACGC GCAGCCCGGA CTCGGTAATG GCGCGCATTG CGCCCAGCGC
PLproC111S  GCACCAACGC GCAGCCCGGA CTCGGTAATG GCGCGCATTG CGCCCAGCGC
 Consensus  GCACCAACGC GCAGCCCGGA CTCGGTAATG GCGCGCATTG CGCCCAGCGC

           3851                                              3900
  PLpro_WT  CATCTGATCG TTGGCAACCA GCATCGCAGT GGGAACGATG CCCTCATTCA
PLproC111S  CATCTGATCG TTGGCAACCA GCATCGCAGT GGGAACGATG CCCTCATTCA
 Consensus  CATCTGATCG TTGGCAACCA GCATCGCAGT GGGAACGATG CCCTCATTCA

           3901                                              3950
  PLpro_WT  GCATTTGCAT GGTTTGTTGA AAACCGGACA TGGCACTCCA GTCGCCTTCC
PLproC111S  GCATTTGCAT GGTTTGTTGA AAACCGGACA TGGCACTCCA GTCGCCTTCC
 Consensus  GCATTTGCAT GGTTTGTTGA AAACCGGACA TGGCACTCCA GTCGCCTTCC

           3951                                              4000
  PLpro_WT  CGTTCCGCTA TCGGCTGAAT TTGATTGCGA GTGAGATATT TATGCCAGCC
PLproC111S  CGTTCCGCTA TCGGCTGAAT TTGATTGCGA GTGAGATATT TATGCCAGCC
 Consensus  CGTTCCGCTA TCGGCTGAAT TTGATTGCGA GTGAGATATT TATGCCAGCC

           4001                                              4050
  PLpro_WT  AGCCAGACGC AGACGCGCCG AGACAGAACT TAATGGGCCC GCTAACAGCG
PLproC111S  AGCCAGACGC AGACGCGCCG AGACAGAACT TAATGGGCCC GCTAACAGCG
 Consensus  AGCCAGACGC AGACGCGCCG AGACAGAACT TAATGGGCCC GCTAACAGCG

           4051                                              4100
  PLpro_WT  CGATTTGCTG GTGACCCAAT GCGACCAGAT GCTCCACGCC CAGTCGCGTA
```

```
PLproC111S  CGATTTGCTG GTGACCCAAT GCGACCAGAT GCTCCACGCC CAGTCGCGTA
 Consensus  CGATTTGCTG GTGACCCAAT GCGACCAGAT GCTCCACGCC CAGTCGCGTA

            4101                                             4150
  PLpro_WT  CCGTCTTCAT GGGAGAAAAT AATACTGTTG ATGGGTGTCT GGTCAGAGAC
PLproC111S  CCGTCTTCAT GGGAGAAAAT AATACTGTTG ATGGGTGTCT GGTCAGAGAC
 Consensus  CCGTCTTCAT GGGAGAAAAT AATACTGTTG ATGGGTGTCT GGTCAGAGAC

            4151                                             4200
  PLpro_WT  ATCAAGAAAT AACGCCGGAA CATTAGTGCA GGCAGCTTCC ACAGCAATGG
PLproC111S  ATCAAGAAAT AACGCCGGAA CATTAGTGCA GGCAGCTTCC ACAGCAATGG
 Consensus  ATCAAGAAAT AACGCCGGAA CATTAGTGCA GGCAGCTTCC ACAGCAATGG

            4201                                             4250
  PLpro_WT  CATCCTGGTC ATCCAGCGGA TAGTTAATGA TCAGCCCACT GACGCGTTGC
PLproC111S  CATCCTGGTC ATCCAGCGGA TAGTTAATGA TCAGCCCACT GACGCGTTGC
 Consensus  CATCCTGGTC ATCCAGCGGA TAGTTAATGA TCAGCCCACT GACGCGTTGC

            4251                                             4300
  PLpro_WT  GCGAGAAGAT TGTGCACCGC CGCTTTACAG GCTTCGACGC CGCTTCGTTC
PLproC111S  GCGAGAAGAT TGTGCACCGC CGCTTTACAG GCTTCGACGC CGCTTCGTTC
 Consensus  GCGAGAAGAT TGTGCACCGC CGCTTTACAG GCTTCGACGC CGCTTCGTTC

            4301                                             4350
  PLpro_WT  TACCATCGAC ACCACCACGC TGGCACCCAG TTGATCGGCG CGAGATTTAA
PLproC111S  TACCATCGAC ACCACCACGC TGGCACCCAG TTGATCGGCG CGAGATTTAA
 Consensus  TACCATCGAC ACCACCACGC TGGCACCCAG TTGATCGGCG CGAGATTTAA

            4351                                             4400
  PLpro_WT  TCGCCGCGAC AATTTGCGAC GGCGCGTGCA GGGCCAGACT GGAGGTGGCA
PLproC111S  TCGCCGCGAC AATTTGCGAC GGCGCGTGCA GGGCCAGACT GGAGGTGGCA
 Consensus  TCGCCGCGAC AATTTGCGAC GGCGCGTGCA GGGCCAGACT GGAGGTGGCA

            4401                                             4450
  PLpro_WT  ACGCCAATCA GCAACGACTG TTTGCCCGCC AGTTGTTGTG CCACGCGGTT
PLproC111S  ACGCCAATCA GCAACGACTG TTTGCCCGCC AGTTGTTGTG CCACGCGGTT
 Consensus  ACGCCAATCA GCAACGACTG TTTGCCCGCC AGTTGTTGTG CCACGCGGTT

            4451                                             4500
  PLpro_WT  GGGAATGTAA TTCAGCTCCG CCATCGCCGC TTCCACTTTT TCCCGCGTTT
PLproC111S  GGGAATGTAA TTCAGCTCCG CCATCGCCGC TTCCACTTTT TCCCGCGTTT
 Consensus  GGGAATGTAA TTCAGCTCCG CCATCGCCGC TTCCACTTTT TCCCGCGTTT

            4501                                             4550
  PLpro_WT  TCGCAGAAAC GTGGCTGGCC TGGTTCACCA CGCGGGAAAC GGTCTGATAA
PLproC111S  TCGCAGAAAC GTGGCTGGCC TGGTTCACCA CGCGGGAAAC GGTCTGATAA
 Consensus  TCGCAGAAAC GTGGCTGGCC TGGTTCACCA CGCGGGAAAC GGTCTGATAA

            4551                                             4600
  PLpro_WT  GAGACACCGG CATACTCTGC GACATCGTAT AACGTTACTG GTTTCACATT
PLproC111S  GAGACACCGG CATACTCTGC GACATCGTAT AACGTTACTG GTTTCACATT
 Consensus  GAGACACCGG CATACTCTGC GACATCGTAT AACGTTACTG GTTTCACATT
```

```
             4601                                               4650
  PLpro_WT   CACCACCCTG AATTGACTCT CTTCCGGGCG CTATCATGCC ATACCGCGAA
PLproC111S   CACCACCCTG AATTGACTCT CTTCCGGGCG CTATCATGCC ATACCGCGAA
 Consensus   CACCACCCTG AATTGACTCT CTTCCGGGCG CTATCATGCC ATACCGCGAA

             4651                                               4700
  PLpro_WT   AGGTTTTGCG CCATTCGATG GTGTCCGGGA TCTCGACGCT CTCCCTTATG
PLproC111S   AGGTTTTGCG CCATTCGATG GTGTCCGGGA TCTCGACGCT CTCCCTTATG
 Consensus   AGGTTTTGCG CCATTCGATG GTGTCCGGGA TCTCGACGCT CTCCCTTATG

             4701                                               4750
  PLpro_WT   CGACTCCTGC ATTAGGAAGC AGCCCAGTAG TAGGTTGAGG CCGTTGAGCA
PLproC111S   CGACTCCTGC ATTAGGAAGC AGCCCAGTAG TAGGTTGAGG CCGTTGAGCA
 Consensus   CGACTCCTGC ATTAGGAAGC AGCCCAGTAG TAGGTTGAGG CCGTTGAGCA

             4751                                               4800
  PLpro_WT   CCGCCGCCGC AAGGAATGGT GCATGCAAGG AGATGGCGCC CAACAGTCCC
PLproC111S   CCGCCGCCGC AAGGAATGGT GCATGCAAGG AGATGGCGCC CAACAGTCCC
 Consensus   CCGCCGCCGC AAGGAATGGT GCATGCAAGG AGATGGCGCC CAACAGTCCC

             4801                                               4850
  PLpro_WT   CCGGCCACGG GGCCTGCCAC CATACCCACG CCGAAACAAG CGCTCATGAG
PLproC111S   CCGGCCACGG GGCCTGCCAC CATACCCACG CCGAAACAAG CGCTCATGAG
 Consensus   CCGGCCACGG GGCCTGCCAC CATACCCACG CCGAAACAAG CGCTCATGAG

             4851                                               4900
  PLpro_WT   CCCGAAGTGG CGAGCCCGAT CTTCCCCATC GGTGATGTCG GCGATATAGG
PLproC111S   CCCGAAGTGG CGAGCCCGAT CTTCCCCATC GGTGATGTCG GCGATATAGG
 Consensus   CCCGAAGTGG CGAGCCCGAT CTTCCCCATC GGTGATGTCG GCGATATAGG

             4901                                               4950
  PLpro_WT   CGCCAGCAAC CGCACCTGTG GCGCCGGTGA TGCCGGCCAC GATGCGTCCG
PLproC111S   CGCCAGCAAC CGCACCTGTG GCGCCGGTGA TGCCGGCCAC GATGCGTCCG
 Consensus   CGCCAGCAAC CGCACCTGTG GCGCCGGTGA TGCCGGCCAC GATGCGTCCG

             4951                                               5000
  PLpro_WT   GCGTAGAGGA TCGAGATCTC GATCCCGCGA AATTAATACG ACTCACTATA
PLproC111S   GCGTAGAGGA TCGAGATCTC GATCCCGCGA AATTAATACG ACTCACTATA
 Consensus   GCGTAGAGGA TCGAGATCTC GATCCCGCGA AATTAATACG ACTCACTATA

             5001                                               5050
  PLpro_WT   GGGGAATTGT GAGCGGATAA CAATTCCCCT CTAGAAATAA TTTTGTTTAA
PLproC111S   GGGGAATTGT GAGCGGATAA CAATTCCCCT CTAGAAATAA TTTTGTTTAA
 Consensus   GGGGAATTGT GAGCGGATAA CAATTCCCCT CTAGAAATAA TTTTGTTTAA

             5051                                               5100
  PLpro_WT   CTTTAAGAAG GAGATATACC ATGGGCAGCA GCCATCATCA TCATCATCAC
PLproC111S   CTTTAAGAAG GAGATATACC ATGGGCAGCA GCCATCATCA TCATCATCAC
 Consensus   CTTTAAGAAG GAGATATACC ATGGGCAGCA GCCATCATCA TCATCATCAC

             5101                                               5150
```

```
  PLpro_WT   AGCAGCGGCC TGGTGCCGCG CGGCAGCCAT CATATGGAGG TCCGCACCAT
PLproC111S   AGCAGCGGCC TGGTGCCGCG CGGCAGCCAT CATATGGAGG TCCGCACCAT
 Consensus   AGCAGCGGCC TGGTGCCGCG CGGCAGCCAT CATATGGAGG TCCGCACCAT

             5151                                              5200
  PLpro_WT   CAAAGTTTTC ACTACTGTGG ATAACATCAA CCTGCATACC CAGGTGGTTG
PLproC111S   CAAAGTTTTC ACTACTGTGG ATAACATCAA CCTGCATACC CAGGTGGTTG
 Consensus   CAAAGTTTTC ACTACTGTGG ATAACATCAA CCTGCATACC CAGGTGGTTG

             5201                                              5250
  PLpro_WT   ACATGTCTAT GACATACGGC CAGCAGTTTG GGCCAACTTA CCTGGACGGC
PLproC111S   ACATGTCTAT GACATACGGC CAGCAGTTTG GGCCAACTTA CCTGGACGGC
 Consensus   ACATGTCTAT GACATACGGC CAGCAGTTTG GGCCAACTTA CCTGGACGGC

             5251                                              5300
  PLpro_WT   GCGGACGTTA CCAAAATTAA ACCGCACAAC TCCCATGAAG GGAAAACCTT
PLproC111S   GCGGACGTTA CCAAAATTAA ACCGCACAAC TCCCATGAAG GGAAAACCTT
 Consensus   GCGGACGTTA CCAAAATTAA ACCGCACAAC TCCCATGAAG GGAAAACCTT

             5301                                              5350
  PLpro_WT   CTACGTACTG CCTAACGATG ACACCCTGCG TGTGGAAGCT TTTGAATACT
PLproC111S   CTACGTACTG CCTAACGATG ACACCCTGCG TGTGGAAGCT TTTGAATACT
 Consensus   CTACGTACTG CCTAACGATG ACACCCTGCG TGTGGAAGCT TTTGAATACT

             5351                                              5400
  PLpro_WT   ACCACACGAC CGACCCGAGT TTCCTTGGTC GTTATATGAG CGCTTTGAAC
PLproC111S   ACCACACGAC CGACCCGAGT TTCCTTGGTC GTTATATGAG CGCTTTGAAC
 Consensus   ACCACACGAC CGACCCGAGT TTCCTTGGTC GTTATATGAG CGCTTTGAAC

             5401                                              5450
  PLpro_WT   CACACCAAAA AATGGAAGTA CCCACAAGTG AACGGTCTGA CTAGCATCAA
PLproC111S   CACACCAAAA AATGGAAGTA CCCACAAGTG AACGGTCTGA CTAGCATCAA
 Consensus   CACACCAAAA AATGGAAGTA CCCACAAGTG AACGGTCTGA CTAGCATCAA

             5451                                              5500
  PLpro_WT   ATGGGCTGAT AACAATTGCT ACCTAGCTAC CGCACTGCTG ACACTGCAAC
PLproC111S   ATGGGCTGAT AACAATTCCT ACCTAGCTAC CGCACTGCTG ACACTGCAAC
 Consensus   ATGGGCTGAT AACAATTcCT ACCTAGCTAC CGCACTGCTG ACACTGCAAC

             5501                                              5550
  PLpro_WT   AGATCGAGCT GAAGTTCAAC CCGCCGGCGT TACAGGATGC TTACTACCGT
PLproC111S   AGATCGAGCT GAAGTTCAAC CCGCCGGCGT TACAGGATGC TTACTACCGT
 Consensus   AGATCGAGCT GAAGTTCAAC CCGCCGGCGT TACAGGATGC TTACTACCGT

             5551                                              5600
  PLpro_WT   GCTCGCGCCG GCGAAGCAGC TAACTTTTGC GCACTGATTC TGGCTTATTG
PLproC111S   GCTCGCGCCG GCGAAGCAGC TAACTTTTGC GCACTGATTC TGGCTTATTG
 Consensus   GCTCGCGCCG GCGAAGCAGC TAACTTTTGC GCACTGATTC TGGCTTATTG

             5601                                              5650
  PLpro_WT   TAACAAAACC GTGGGCGAAC TGGGTGATGT TCGTGAAACC ATGAGTTACC
PLproC111S   TAACAAAACC GTGGGCGAAC TGGGTGATGT TCGTGAAACC ATGAGTTACC
```

```
 Consensus   TAACAAAACC GTGGGCGAAC TGGGTGATGT TCGTGAAACC ATGAGTTACC

             5651                                             5700
  PLpro_WT   TTTTCCAGCA CGCTAATCTG GACTCATGCA AACGCGTCCT GAACGTGGTA
PLproC111S   TTTTCCAGCA CGCTAATCTG GACTCATGCA AACGCGTCCT GAACGTGGTA
 Consensus   TTTTCCAGCA CGCTAATCTG GACTCATGCA AACGCGTCCT GAACGTGGTA

             5701                                             5750
  PLpro_WT   TGTAAAACCT GCGGTCAGCA GCAAACCACA CTCAAAGGCG TTGAAGCCGT
PLproC111S   TGTAAAACCT GCGGTCAGCA GCAAACCACA CTCAAAGGCG TTGAAGCCGT
 Consensus   TGTAAAACCT GCGGTCAGCA GCAAACCACA CTCAAAGGCG TTGAAGCCGT

             5751                                             5800
  PLpro_WT   TATGTATATG GGCACTCTGA GTTATGAGCA GTTTAAAAAA GGTGTGCAGA
PLproC111S   TATGTATATG GGCACTCTGA GTTATGAGCA GTTTAAAAAA GGTGTGCAGA
 Consensus   TATGTATATG GGCACTCTGA GTTATGAGCA GTTTAAAAAA GGTGTGCAGA

             5801                                             5850
  PLpro_WT   TCCCGTGTAC TTGCGGCAAA CAGGCTACCA AGTATCTGGT TCAGCAGGAA
PLproC111S   TCCCGTGTAC TTGCGGCAAA CAGGCTACCA AGTATCTGGT TCAGCAGGAA
 Consensus   TCCCGTGTAC TTGCGGCAAA CAGGCTACCA AGTATCTGGT TCAGCAGGAA

             5851                                             5900
  PLpro_WT   TCTCCATTCG TAATGATGAG CGCACCACCG GCGCAGTACG AACTCAAACA
PLproC111S   TCTCCATTCG TAATGATGAG CGCACCACCG GCGCAGTACG AACTCAAACA
 Consensus   TCTCCATTCG TAATGATGAG CGCACCACCG GCGCAGTACG AACTCAAACA

             5901                                             5950
  PLpro_WT   CGGGACTTTT ACTTGCGCGT CTGAATACAC AGGTAATTAC CAGTGTGGTC
PLproC111S   CGGGACTTTT ACTTGCGCGT CTGAATACAC AGGTAATTAC CAGTGTGGTC
 Consensus   CGGGACTTTT ACTTGCGCGT CTGAATACAC AGGTAATTAC CAGTGTGGTC

             5951                                             6000
  PLpro_WT   ACTACAAACA TATTACGTCC AAAGAAACTC TCTATTGCAT CGACGGCGCT
PLproC111S   ACTACAAACA TATTACGTCC AAAGAAACTC TCTATTGCAT CGACGGCGCT
 Consensus   ACTACAAACA TATTACGTCC AAAGAAACTC TCTATTGCAT CGACGGCGCT

             6001                                             6050
  PLpro_WT   CTGCTGACCA AATCGAGCGA ATACAAAGGC CCTATCACGG ATGTCTTCTA
PLproC111S   CTGCTGACCA AATCGAGCGA ATACAAAGGC CCTATCACGG ATGTCTTCTA
 Consensus   CTGCTGACCA AATCGAGCGA ATACAAAGGC CCTATCACGG ATGTCTTCTA

             6051                                             6100
  PLpro_WT   CAAAGAGAAC TCGTACACGA CCACTATCAA ACCGGTTACG TATTAGTCGA
PLproC111S   CAAAGAGAAC TCGTACACGA CCACTATCAA ACCGGTTACG TATTAGTCGA
 Consensus   CAAAGAGAAC TCGTACACGA CCACTATCAA ACCGGTTACG TATTAGTCGA

             6101                                             6150
  PLpro_WT   CAAGCTTGCG GCCGCACTCG AGCACCACCA CCACCACCAC TGAGATCCGG
PLproC111S   CAAGCTTGCG GCCGCACTCG AGCACCACCA CCACCACCAC TGAGATCCGG
 Consensus   CAAGCTTGCG GCCGCACTCG AGCACCACCA CCACCACCAC TGAGATCCGG
```

```
                6151                                                  6200
   PLpro_WT    CTGCTAACAA AGCCCGAAAG GAAGCTGAGT TGGCTGCTGC CACCGCTGAG
 PLproC111S    CTGCTAACAA AGCCCGAAAG GAAGCTGAGT TGGCTGCTGC CACCGCTGAG
  Consensus    CTGCTAACAA AGCCCGAAAG GAAGCTGAGT TGGCTGCTGC CACCGCTGAG

                6201                                                  6250
   PLpro_WT    CAATAACTAG CATAACCCCT TGGGGCCTCT AAACGGGTCT TGAGGGGTTT
 PLproC111S    CAATAACTAG CATAACCCCT TGGGGCCTCT AAACGGGTCT TGAGGGGTTT
  Consensus    CAATAACTAG CATAACCCCT TGGGGCCTCT AAACGGGTCT TGAGGGGTTT

                6251                            6279
   PLpro_WT    TTTGCTGAAA GGAGGAACTA TATCCGGAT
 PLproC111S    TTTGCTGAAA GGAGGAACTA TATCCGGAT
  Consensus    TTTGCTGAAA GGAGGAACTA TATCCGGAT
```

7. Mass spectra of native PL^{pro} in complex with compound 34

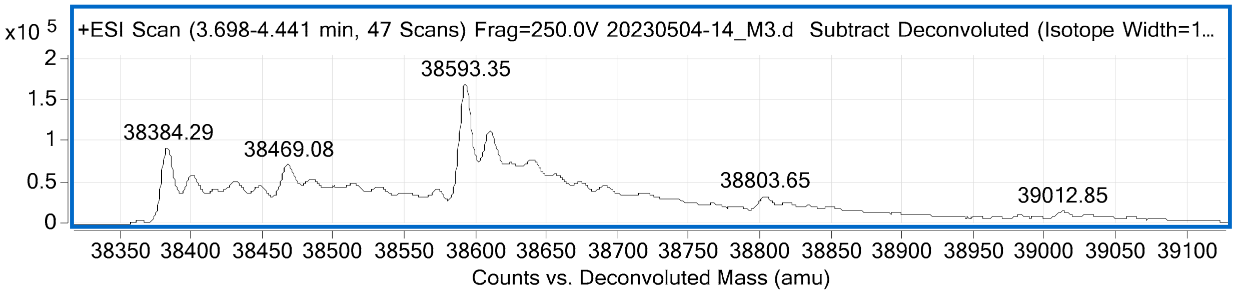

Figure S21. Mass spectra of native PL^{pro} in complex with compound **34**. The difference compared to the molecular weight of native PL^{pro} is equal to 210.11 Da. MW (native PL^{pro}) – MW (PL^{pro}-**34** complex) = 38593.35-38384.29 = 210.28) (corresponds to MW(**34**)/2 = 420.55/2 = 210.28).

References:

[1] B. Grybaitė, I. Jonuškienė, R. Vaickelionienė, V. Mickevičius, Synthesis, transformation and antibacterial activity of new N,N-disubstituted 2-aminothiazole derivatives, Chemija. (2017).

[2] B. Grybaitė, R. Vaickelionienė, M. Stasevych, O. Komarovska-Porokhnyavets, V. Novikov, V. Mickevičius, Synthesis, Transformation of 3-[(4-Arylthiazol-2-yl)(p-tolyl)amino]propanoic Acids, Bis(thiazol-5-yl)phenyl-, Bis(thiazol-5-yl)methane Derivatives, and Their Antimicrobial Activity, HETEROCYCLES. 96 (2018) 86. https://doi.org/10.3987/COM-17-13833.

[3] I. Tumosienė, E. Jakienė, Z.J. Beresnevičius, G. Mikulskienė, Synthesis and properties of dihydrazides of N-phenyl-and N-(4-methylphenyl)-N-carboxyethyl-β-alanines, Cheminė Technologija. 3 (2006) 58–64.

[4] B. Grybaitė, R. Vaickelionienė, M. Stasevych, O. Komarovska-Porokhnyavets, K. Kantminienė, V. Novikov, V. Mickevičius, Synthesis and Antimicrobial Activity of Novel Thiazoles with Reactive Functional Groups, ChemistrySelect. 4 (2019) 6965–6970. https://doi.org/10.1002/slct.201900679.

[5] B. Sapijanskaitė-Banevič, B. Grybaitė, R. Vaickelionienė, I. Bružaitė, Synthesis, transformation and preliminary bioassay of 3-(thiazol-2-yl(p-tolyl)amino)propanoic acid derivatives, Chemija. 34 (2023). https://doi.org/10.6001/chemija.2023.34.1.7.

[6] R. Minickaitė, B. Grybaitė, R. Vaickelionienė, P. Kavaliauskas, V. Petraitis, R. Petraitienė, I. Tumosienė, I. Jonuškienė, V. Mickevičius, Synthesis of Novel Aminothiazole Derivatives as Promising Antiviral, Antioxidant and Antibacterial Candidates, Int. J. Mol. Sci. 23 (2022) 7688. https://doi.org/10.3390/ijms23147688.

[7] K. Skrickus, J. Šiugždaitė, R. Lelešiu, R. Anusevičius, B. Grybaitė, R. Vaickelionienė, V. Mickevičius, Synthesis, characterization and antibacterial assays of novel N,N1-disubstituted 2,2'-dithiodianiline derivatives, Chem. Sel. 8 (n.d.) e202300332. https://doi.org/10.1002/slct.202300332.
